# Hamiltonian Dynamics and Fundamental Phenomena in Biophysics: A Review

**DOI:** 10.3390/e28080928

**Published:** 2026-08-19

**Authors:** Matteo Gori, Roberto Franzosi, Giulio Pettini, Marco Pettini

**Affiliations:** 1 Department of Physics and Sciences of Materials, University of Luxembourg, L-1511 Luxembourg, Luxembourg; matteo.gori@uni.lu; 2 Dipartimento di Scienze Fisiche, della Terra e dell’Ambiente, University of Siena, Via Roma 56, 53100 Siena, Italy; roberto.franzosi@ino.it; 3 Dipartimento di Fisica, University of Firenze, Via G. Sansone 1, 50019 Sesto Fiorentino, Italy; pettini@fi.unifi.it; 4 I.N.F.N., Sezione di Firenze, Via G. Sansone 1, 50019 Sesto Fiorentino, Italy; 5 Department of Physics, Aix-Marseille University, 13288 Marseille, France; 6 CNRS Centre de Physique Théorique UMR7332, 13288 Marseille, France; 7 Quantum Biology Lab, Howard University, 2400 6th St NW, Washington, DC 20059, USA

**Keywords:** Fröhlich collective oscillations, long-range electrodynamic intermolecular forces, THz spectroscopy

## Abstract

We review a theoretical and experimental programme with the aim of understanding two intimately related fundamental phenomena in biophysics: (i) the classical analogue of Fröhlich phonon condensation in macromolecules driven out of thermal equilibrium and (ii) the consequent activation of long-range resonant electrodynamic intermolecular forces. Both phenomena are underpinned by explicit Hamiltonian models. The first is derived by applying the time-dependent variational principle (TDVP) to the quantum Wu–Austin model, producing a fully classical Hamiltonian in action-angle variables whose nonlinear rate equations exhibit a nonequilibrium phase transition: the channelling of supplied energy into the lowest-frequency collective mode. The second is grounded in a classical electrodynamic Hamiltonian for two coupled oscillating dipoles whose normal-mode structure predicts long-range (∼$1/r^{3}$) resonant interactions, absent at thermal equilibrium but activated by out-of-equilibrium collective oscillations. We also discuss a complementary Hamiltonian approach that connects Fröhlich’s rate equations directly to Hamilton’s equations of motion, clarifying the role of bath-mediated nonlinear coupling and the conditions for strong condensation at room temperature. In addition, the TDVP is applied to a Davydov–Holstein–Fröhlich Hamiltonian describing electron–phonon motion along the backbone of a specific DNA sequence and its cognate restriction enzyme, EcoRI: the time-domain Fourier cross-spectrum of the resulting electron currents exhibits a sharp co-resonance peak for the canonical recognition sequence that disappears upon randomisation, providing a sequence-specific electrodynamic signature of DNA–protein recognition. Experimental evidence from THz near-field spectroscopy, fluorescence correlation spectroscopy, and direct observation of protein clustering is reviewed in relation to these theoretical predictions. The results establish a coherent physical picture suggesting that metabolic energy supply can play a role in driving macromolecules into coherently oscillating states that activate selective, distance-reaching electrodynamic forces capable of contributing to the organisation of biochemical reactions in living matter.

## 1. Introduction

A fundamental challenge in biophysics is to understand how biochemical reactions in living cells proceed with the speed and selectivity observed experimentally, given the crowded, thermally noisy environment of the cytoplasm. Standard models invoke short-range forces and electrostatic, van der Waals, hydrophobic, and diffusion-driven random encounters. These mechanisms are undoubtedly operative, but they do not obviously account for the remarkably high efficiency and rapidity with which cognate molecular partners find one another among thousands of competing species in a sub-micron space [1,2,3].

A minority but persistent view in theoretical biophysics, originating with H. Fröhlich in the late 1960s [4,5,6,7], proposes that biological macromolecules driven out of thermodynamic equilibrium by metabolic energy could undergo a collective ordering transition, a *phonon condensation,* in which the input energy concentrates into the lowest-frequency vibrational mode of a macromolecule, producing a coherent giant oscillating dipole moment. This coherent oscillation was predicted to activate resonant, selective, long-range electrodynamic forces between macromolecules vibrating at the same frequency [6], forces that could accelerate and direct molecular recognition processes.

Establishing this mechanism on firm Hamiltonian and experimental footing matters beyond the immediate biophysics of Fröhlich condensation: it would supply a physically explicit, falsifiable alternative to purely stochastic pictures of molecular recognition, with direct relevance to how enzymes locate cognate substrates, how homologous DNA sequences find one another, and more generally to the long-standing question of how living matter achieves reaction rates and specificities that diffusion-and-collision kinetics alone struggle to explain. The present review is intended to make this significance explicit for readers outside the immediate field, alongside the technical derivations that follow.

Three key obstacles have historically limited the impact of this programme. First, the original quantum Wu–Austin microscopic Hamiltonian [8,9] suffers from an instability that requires stabilisation. Second, the connection between the rate equation level and direct Hamiltonian dynamics had not been established in a classical framework accessible to modern molecular dynamics. Third, and most critically, no experimental evidence for out-of-equilibrium collective oscillations of macromolecules existed until recently.

All these obstacles have now been overcome. The present work reviews the following pivotal topics: *(i)* A classical Hamiltonian in action-angle variables is derived from the quantum Wu–Austin model via the TDVP applied to product of coherent states (Section 2, Section 3 and Section 4). Nonlinear rate equations follow from the Koopman–von Neumann (KvN) reformulation of the Liouville equation. Their stationary solutions exhibit classical phonon condensation [10]. *(ii)* A classical electrodynamic Hamiltonian for two coupled oscillating dipoles is analysed via normal-mode theory (Section 5). The result is a long-range (∼$1/r^{3}$) interaction potential, active only out of thermal equilibrium [11]. *(iii)* Position-space classical Hamiltonian dynamics, closer to standard molecular dynamics, is simulated directly (Section 6), recovering the outcomes of the Fröhlich rate equations and showing that the formation of strong condensates at room temperature can be found under resonant bath-mediated coupling [12]. *(iv)* The TDVP is applied to a Davydov–Holstein–Fröhlich Hamiltonian for electron–phonon motion along a specific DNA sequence and its cognate restriction enzyme EcoRI (Section 7), and the associate dynamics yields a sharp cross-spectral co-resonance between the electron currents of the two molecules, which appears for the canonical recognition sequence and disappears upon randomisation [13].

The experimental evidence is reviewed in Section 8. Section 9 gives an outlook of future developments. We stress that this article is a review; with the exception of the connections drawn between the different formalisms and the side-by-side presentation of the two channels in Section 9, none of the derivations, simulations, or experimental data presented below are original to the present work. Every theoretical result reproduced here was first derived in Refs. [10,11,12,13,14], and every experimental result was first reported in Refs. [10,15]; corresponding citations are given at the points where each result is introduced, and figure captions throughout indicate the source reference explicitly. The review’s own contribution is the unification of these results under the common TDVP-dequantization framework used in Section 3 and Section 7, and the synthesis of the vibrational and electronic channels as two complementary aspects of a single physical picture.

## 2. The Wu–Austin Quantum Model and the Classical Limit

### 2.1. Physical Picture

Fröhlich [4,5] modelled a macromolecule as an open system: an ensemble of coupled vibrational modes simultaneously in contact with a thermal bath at temperature $T_{B}$ and with an energy source at the effective temperature, $T_{S}\gg T_{B}$. He argued that, when the energy-input rate exceeds a threshold, the system undergoes a nonequilibrium phase transition: instead of distributing the supplied energy among all modes (equipartition), it channels nearly all of it into the lowest-frequency mode, producing a macroscopic coherent oscillation.

#### 2.1.1. Fröhlich Rate Equations and Condensation

At the phenomenological level, Fröhlich described the dynamics in terms of the occupation numbers, $n_{k}\left(t\right)=\langle {\hat{a}}_{\omega_{k}}^{†}{\hat{a}}_{\omega_{k}}\rangle$, of the vibrational modes [7]. Consider a set of *z* polar modes with frequencies $\omega_{1}\leq \omega_{k}\leq \omega_{z}$, k=1,…,z, coupled to a heat bath at inverse temperature $\beta_{B}=1/k_{B}T_{B}$ and driven by an external source that injects quanta into mode *k* at rate $s_{k}$. Two classes of bath-mediated processes are retained.

*First-order* processes exchange a single quantum between mode *k* and the bath, tending to restore thermal equilibrium. *Second-order* processes transfer excitations between two different system modes, *k* and *j*, with the bath absorbing or supplying the energy difference; crucially, they conserve the total number of system quanta, $\sum_{k}{\dot{n}}_{k}^{\left(2\right)}=0$. Denoting by φ the linear relaxation coefficient and by χ the nonlinear intermode coupling, the complete rate equations are (for k=1,…,z)

(1) $${\dot{n}}_{k}=s_{k}-\varphi \;\left[n_{k}\;e^{\beta_{B}\hbar \omega_{k}}-\left(n_{k}+1\right)\right]-\chi \sum_{j\neq k}\;\left[n_{k}\left(1+n_{j}\right)\;e^{\beta_{B}\hbar \omega_{k}}-n_{j}\left(1+n_{k}\right)\;e^{\beta_{B}\hbar \omega_{j}}\right].$$

The first term injects energy from the source; the second drives each mode toward thermal equilibrium with the bath; the third redistributes quanta across the spectrum without altering their total number.

##### Effective Chemical Potential

This nonlinear redistribution is the key to the Fröhlich effect. Because downward transitions in frequency are statistically favoured by the Bose factors $\left(1+n_{j}\right)$, pumped energy is not simply equipartitioned. Once the total input rate, $S=\sum_{k}s_{k}$, exceeds a threshold set by the competition between pumping, dissipation, and nonlinear scattering, the excess population can no longer be absorbed by the higher-frequency modes and accumulates at the bottom of the spectrum. In the stationary regime, one introduces an effective nonequilibrium chemical potential, μ, defined self-consistently by

(2) $$e^{-\beta_{B}\mu }=\frac{\varphi +\chi \sum_{j}\left(1+n_{j}\right)}{\varphi +\chi \sum_{j}n_{j}\;e^{\beta_{B}\hbar \omega_{j}}},$$

which satisfies $0\leq \mu <\hbar \omega_{1}$ whenever S>0. The formal stationary solution then takes the Bose-like form

(3) $$n_{k}=\left(1+\frac{\varphi }{\chi }\frac{s_{k}}{S}\left(1-e^{-\beta_{B}\mu }\right)\right)\frac{1}{e^{\beta_{B}\left(\hbar \omega_{k}-\mu \right)}-1},$$

so that, as *S* increases, μ approaches $\hbar \omega_{1}$ from below and $n_{1}$ grows without bound while all other occupations remain comparatively limited.

##### Fröhlich Condensation

The Fröhlich condensation phenomenon is as follows: a nonequilibrium ordered state in which a single low-frequency polar mode becomes macroscopically populated and behaves as a collective coherent oscillation of the macromolecule. The analogy with Bose–Einstein condensation is instructive: in both cases, the discrete sum over modes cannot be replaced by an integral once μ saturates at the band edge, forcing a macroscopic occupation of the ground mode. The mechanism is, however, intrinsically, a nonequilibrium one. In an equilibrium Bose gas, the condensed state is reached by lowering the temperature; here, it is reached by maintaining a sufficiently strong energy throughput in an open, dissipative system at fixed $T_{B}$. When the nonlinear coupling vanishes (χ=0), Equation (2) gives μ=0 and Equation (3) reduces to a modified Planck distribution with no condensation; it is therefore the nonlinear bath-mediated intermode scattering that is the essential ingredient.

The predicted coherent oscillation lies in the sub-THz range, $\nu ∼10^{11}$ Hz, set by the acoustic-like breathing vibrations of proteins or membrane sections of a linear size of $\ell ∼10^{-6}$ cm, with effective sound velocity, $v∼10^{5}$ cm/s [7]. Once the lowest polar mode is macroscopically occupied, it acts as an oscillating giant dipole; cell water with small ions in solution, with a typical Debye length of 10 Å, screens static charges efficiently, but does not screen oscillating fields at these frequencies. Two such coherently oscillating dipoles at separation *R* interact via a frequency-selective, attractive potential that, in the near-resonance limit, falls off as [7]

(4) $$I≃-U\;\frac{e^{2}{\left(z_{e}z_{s}\right)}^{1/2}}{M\omega_{e}^{2}\;R^{3}}\;\propto \;-\frac{1}{R^{3}},$$

where $U=\hbar n_{-}\omega_{e}$ is the excitation energy, $z_{e},z_{s}$ are the numbers of coherently oscillating charges, *M* is their mass, and $n_{-}$ is the occupation of the lower normal mode of the coupled pair. This long-range force is proposed as the mechanism for frequency-selective recognition between enzymes and their substrates and for the attraction of homologous chromosomes during meiosis [7].

#### 2.1.2. Microscopic Derivation: Wu–Austin Hamiltonian

A microscopic quantum Hamiltonian from which Fröhlich’s rate equations can be derived was given by Wu and Austin [8,9]. The system consists of three sets of quantum harmonic oscillators: normal modes of the macromolecule (frequencies of $\omega_{i}\in I_{\mathrm{sys}}$; operators, ${\hat{a}}_{\omega_{i}}$, ${\hat{a}}_{\omega_{i}}^{†}$); a thermal bath at $T_{B}$ (frequencies of $\Omega_{j}\in I_{\mathrm{bth}}$; operators, ${\hat{b}}_{\Omega_{j}}$, ${\hat{b}}_{\Omega_{j}}^{†}$); and an energy source modelled as a second bath at $T_{S}\gg T_{B}$ (frequencies of $\Omega_{k}^{\prime }\in I_{\mathrm{src}}$; operators, ${\hat{c}}_{\Omega_{k}^{\prime }}$, ${\hat{c}}_{\Omega_{k}^{\prime }}^{†}$). The total Hamiltonian is ${\hat{H}}_{\mathrm{Tot}}={\hat{H}}_{0}+{\hat{H}}_{\mathrm{int}}$, where

(5) $${\hat{H}}_{0}=\sum_{\omega_{i}}\hbar \omega_{i}\;{\hat{a}}_{\omega_{i}}^{†}{\hat{a}}_{\omega_{i}}+\sum_{\Omega_{j}}\hbar \Omega_{j}\;{\hat{b}}_{\Omega_{j}}^{†}{\hat{b}}_{\Omega_{j}}+\sum_{\Omega_{k}^{\prime }}\hbar \Omega_{k}^{\prime }\;{\hat{c}}_{\Omega_{k}^{\prime }}^{†}{\hat{c}}_{\Omega_{k}^{\prime }},$$

the interaction contains the Wu–Austin trilinear couplings

(6) $${\hat{H}}_{\mathrm{int}}^{\mathrm{WA}}=\sum_{\omega_{i},\Omega_{j}}\eta_{\omega_{i}\Omega_{j}}\;{\hat{a}}_{\omega_{i}}^{†}{\hat{b}}_{\Omega_{j}}+\sum_{\omega_{i},\Omega_{k}^{\prime }}\xi_{\omega_{i}\Omega_{k}^{\prime }}\;{\hat{a}}_{\omega_{i}}^{†}{\hat{c}}_{\Omega_{k}^{\prime }}+\sum_{\omega_{i},\omega_{j},\Omega_{k}}\chi_{\omega_{i}\omega_{j}\Omega_{k}}\;{\hat{a}}_{\omega_{i}}^{†}{\hat{a}}_{\omega_{j}}{\hat{b}}_{\Omega_{k}}^{†}+H.c.,$$

supplemented by a stabilising quartic term [16]

(7) $${\hat{H}}_{\mathrm{int}}^{Q}=\sum_{\omega_{i},\omega_{j},\omega_{k},\omega_{l}}\left[\kappa_{\omega_{i}\omega_{j}\omega_{k}\omega_{l}}^{\left(1\right)}\;{\hat{a}}_{\omega_{i}}^{†}{\hat{a}}_{\omega_{j}}^{†}{\hat{a}}_{\omega_{k}}{\hat{a}}_{\omega_{l}}+\kappa_{\omega_{i}\omega_{j}\omega_{k}\omega_{l}}^{\left(2\right)}\;{\hat{a}}_{\omega_{i}}^{†}{\hat{a}}_{\omega_{j}}^{†}{\hat{a}}_{\omega_{k}}^{†}{\hat{a}}_{\omega_{l}}+H.c.\right].$$

The quartic term removes the pathology of the pure Wu–Austin model and corresponds physically to anharmonic interactions among the macromolecular modes. Fröhlich’s phenomenological rate, Equation (1), is recovered from this Hamiltonian by applying the quantum Liouville–von Neumann equation, $i\hbar \;\partial_{t}\hat{\rho }=\left[{\hat{H}}_{\mathrm{Tot}},\hat{\rho }\right]$, to the full density matrix, $\hat{\rho }$, tracing over the bath and source degrees of freedom in the Born–Markov approximation, and identifying $n_{k}=\mathrm{Tr}\;\left({\hat{a}}_{\omega_{k}}^{†}{\hat{a}}_{\omega_{k}}\;{\hat{\rho }}_{\mathrm{sys}}\right)$. The trilinear terms in ${\hat{H}}_{\mathrm{int}}^{\mathrm{WA}}$ generate the linear relaxation (φ) and the pumping ($s_{k}$), while the trilinear system–bath coupling, $\chi_{\omega_{i}\omega_{j}\Omega_{k}}$, produces the nonlinear intermode redistribution term (χ) in (1); the quartic term, ${\hat{H}}_{\mathrm{int}}^{Q}$, ensures the thermodynamic stability of the condensed state.

#### 2.1.3. Why a Classical Description Suffices

At room temperature (T≈300 K) and at the sub-THz frequencies relevant to the collective protein vibrations (ω/2π∼0.1–1 THz), $k_{B}T/\hbar \omega \gg 1$, so the field is in the classical regime and the Bose–Einstein mean occupation number, ${\langle n\rangle }_{\mathrm{BE}}$, is well approximated by the Boltzmann estimate, $k_{B}T/\hbar \omega$, ranging from ≈6 at 1 THz to ≈62 at 0.1 THz. When driven out of equilibrium, the effective temperature of each mode increases further, making the classical approximation even better. A classical framework is therefore not only legitimate but natural, and it connects directly to the language of the molecular dynamics simulation.

## 3. Dequantization via the Time-Dependent Variational Principle

The time-dependent variational principle (TDVP) [17,18,19] extracts a classical Hamiltonian from a quantum one by restricting dynamics to a finite-dimensional manifold of trial states, $|\psi (x_{1},\ldots ,x_{N})\rangle$, parametrised by real variables, $\left\{x_{i}\left(t\right)\right\}$. The equations of motion follow from

(8) $$\delta S=0,\;S=\int_{0}^{t}L\left(\psi ,\bar{\psi }\right)\;dt^{\prime },$$

with the Lagrangian

(9) $$L=i\hbar \;\frac{\langle \psi |\dot{\psi }\rangle -\langle \dot{\psi }|\psi \rangle }{2\langle \psi |\psi \rangle }-\frac{\langle \psi |{\hat{H}}_{\mathrm{Tot}}|\psi \rangle }{\langle \psi |\psi \rangle }.$$

The resulting classical Hamiltonian is simply the expectation value, $H_{\mathrm{Tot}}=\langle \psi |{\hat{H}}_{\mathrm{Tot}}|\psi \rangle$. This procedure has been promoted as a “dequantization” method, a kind of inverse of geometric quantization [19]. Since ${\hat{H}}_{\mathrm{Tot}}$ is built from bosonic creation and annihilation operators, the natural trial states are products of coherent states over all modes in $I_{S}=I_{\mathrm{sys}}\cup I_{\mathrm{bth}}\cup I_{\mathrm{src}}$:

(10) $$|\Psi \left(t\right)\rangle =\underset{\omega_{i}\in I_{\mathrm{sys}}}{⨂}|z_{\omega_{i}}\left(t\right)\rangle ⊗\underset{\Omega_{j}\in I_{\mathrm{bth}}}{⨂}|z_{\Omega_{j}}\left(t\right)\rangle ⊗\underset{\Omega_{k}^{\prime }\in I_{\mathrm{src}}}{⨂}|z_{\Omega_{k}^{\prime }}\left(t\right)\rangle ,$$

where $|z\rangle =e^{-{\left|z\right|}^{2}/2}\sum_{k=0}^{\infty }z^{k}/\sqrt{k!}\;|k\rangle$ satisfies $\langle \hat{n}\rangle ={\left|z\right|}^{2}$. Writing $z_{i}=n_{i}^{1/2}e^{-i\theta_{i}}$ introduces real pairs $\left(n_{i},\theta_{i}\right)$ for every mode. A direct computation of the symplectic tensor $W_{ij}=\partial_{i}w_{j}-\partial_{j}w_{i}$, with $w_{i}=i\hbar \langle \psi |\partial_{x_{i}}\psi \rangle$, yields

(11) $$W_{n_{i}\theta_{k}}=-W_{\theta_{k}n_{i}}=\hbar \;\delta_{ik},\;W_{\theta_{i}\theta_{k}}=W_{n_{i}n_{k}}=0.$$

Hence, $J_{\omega_{i}}\equiv \hbar n_{\omega_{i}}$ and $\theta_{\omega_{i}}$ are *canonically conjugated* variables, $\left\{J_{\omega_{i}},\;\theta_{\omega_{k}}\right\}=\delta_{ik}$. The action $J_{\omega }=\hbar \langle {\hat{n}}_{\omega }\rangle$ equals *ℏ* times the mean occupation number.

### 3.1. The Classical Hamiltonian in Action-Angle Variables

Evaluating $H_{\mathrm{Tot}}=\langle \Psi |{\hat{H}}_{\mathrm{Tot}}|\Psi \rangle$ in $\left(J_{\omega },\theta_{\omega }\right)$ variables, one finds [10]

(12) $$\begin{array}{cc} H_{\mathrm{Tot}}\; & =\sum_{\omega_{i}}\omega_{i}J_{\omega_{i}}+\sum_{\Omega_{j}}\Omega_{j}J_{\Omega_{j}}+\sum_{\Omega_{k}^{\prime }}\Omega_{k}^{\prime }J_{\Omega_{k}^{\prime }}+\sum_{\omega_{i},\Omega_{j}}\eta_{\omega_{i}\Omega_{j}}J_{\omega_{i}}^{1/2}J_{\Omega_{j}}^{1/2}\cos\left(\theta_{\omega_{i}}-\theta_{\Omega_{j}}\right)\\ \; & \;+\sum_{\omega_{i},\Omega_{k}^{\prime }}\xi_{\omega_{i}\Omega_{k}^{\prime }}J_{\omega_{i}}^{1/2}J_{\Omega_{k}^{\prime }}^{1/2}\cos\left(\theta_{\omega_{i}}-\theta_{\Omega_{k}^{\prime }}\right)+\sum_{\omega_{i},\omega_{j},\Omega_{k}}\chi_{\omega_{i}\omega_{j}\Omega_{k}}J_{\omega_{i}}^{1/2}J_{\omega_{j}}^{1/2}J_{\Omega_{k}}^{1/2}\cos\left(\theta_{\omega_{i}}-\theta_{\omega_{j}}+\theta_{\Omega_{k}}\right)\\ \; & \;+\sum_{\omega_{i},\omega_{j},\omega_{k},\omega_{l}}J_{\omega_{i}}^{1/2}J_{\omega_{j}}^{1/2}J_{\omega_{k}}^{1/2}J_{\omega_{l}}^{1/2}\times \left[\kappa_{\ldots }^{\left(1\right)}\cos\left(\theta_{\omega_{i}}+\theta_{\omega_{j}}-\theta_{\omega_{k}}-\theta_{\omega_{l}}\right)+\kappa_{\ldots }^{\left(2\right)}\cos\left(\theta_{\omega_{i}}+\theta_{\omega_{j}}+\theta_{\omega_{k}}-\theta_{\omega_{l}}\right)\right]. \end{array}$$

This is a fully classical, canonical Hamiltonian, retaining the complete structure of the quantum model. Hamilton’s equations ${\dot{J}}_{\omega }=-\partial H/\partial \theta_{\omega }$, ${\dot{\theta }}_{\omega }=\partial H/\partial J_{\omega }$ govern all degrees of freedom.

### 3.2. Classical Rate Equations via the Koopman–Von Neumann Formalism

Rather than integrating Hamilton’s equations with an explicit bath, one works with the phase-space probability density $\rho (\left\{\left(J_{\omega },\theta_{\omega }\right)\right\};t)$, satisfying the Liouville equation

(13) $$\frac{\partial \rho }{\partial t}=\left\{H_{\mathrm{Tot}},\;\rho \right\}\equiv -i{\hat{L}}_{H}\rho .$$

The Koopman–von Neumann (KvN) formalism [20,21] rewrites this as a Schrödinger-like equation, $i\;\partial_{t}\psi ={\hat{L}}_{H}\psi$, $\rho ={\left|\psi \right|}^{2}$, on the Hilbert space, $L^{2}\left(\Lambda \right)$, allowing interaction picture perturbation theory to be applied directly to the classical problem. In this framework, the action variables $J_{\omega_{i}}=\hbar n_{\omega_{i}}$ play the role of the classical counterparts of the quantum occupation numbers, and the conjugate angles, $\theta_{\omega_{i}}$, are the corresponding phases. Decomposing ${\hat{L}}_{H}={\hat{L}}_{H_{0}}+{\hat{L}}_{H_{\mathrm{int}}}$ and treating ${\hat{L}}_{H_{\mathrm{int}}}$ as a time-dependent perturbation adiabatically switched on from $t_{0}=0$, one works in the interaction picture and expands to second order. Averaging over the thermal bath’s initial conditions, compatible with temperatures $T_{B}$ (bath) and $T_{S}\gg T_{B}$ (external source), then yields closed rate equations for $\langle J_{\omega_{i}}\rangle$, in full analogy with the quantum derivation of Fröhlich’s equations via time-dependent perturbation theory [8,9].

### 3.3. Classical Fröhlich-like Rate Equations

The result of the second-order KvN expansion [10] is

(14) $$\frac{d\langle J_{\omega_{i}}\rangle }{dt}=s_{\omega_{i}}+b_{\omega_{i}}\;\left(\frac{k_{B}T_{B}}{\omega_{i}}-\langle J_{\omega_{i}}\rangle \right)+\sum_{\omega_{j}\neq \omega_{i}}c_{\omega_{i}\omega_{j}}\left[\langle J_{\omega_{j}}\rangle -\langle J_{\omega_{i}}\rangle +\frac{\omega_{j}-\omega_{i}}{k_{B}T_{B}}\langle J_{\omega_{i}}\rangle \langle J_{\omega_{j}}\rangle \right]+\left(\mathrm{quartic}\right),$$

where $s_{\omega_{i}}>0$ is the energy-input rate from the external source, $b_{\omega_{i}}$ is the linear system–bath coupling constant (with a characteristic thermalisation timescale of $\tau_{\mathrm{therm}}\approx b_{\omega_{i}}^{-1}$), and $c_{\omega_{i}\omega_{j}}$ encodes the nonlinear mode–mode coupling mediated by the bath. The quartic correction terms, absent in the original Wu–Austin model but necessary to bound the energy from below [16], involve the coupling constants, $\kappa_{\omega_{i}\omega_{j}\omega_{k}\omega_{l}}^{\left(1,2,3\right)}$, of the anharmonic interaction among normal modes [10].

This equation has the following key properties. At equilibrium ($s_{\omega_{i}}=0$), the unique stationary solution is $\langle J_{\omega_{i}}\rangle =k_{B}T_{B}/\omega_{i}$ (energy equipartition), since $y_{\omega_{i}}=\omega_{i}\langle J_{\omega_{i}}\rangle /\left(k_{B}T_{B}\right)=1$ for all modes. Equation (14) is functionally identical to Fröhlich’s original quantum rate equations, with $\langle J_{\omega_{i}}\rangle$ replacing the occupation number, $\langle {\hat{n}}_{\omega_{i}}\rangle$; this functional identity is the classical counterpart of the Bose-like condensation mechanism. As $s_{\omega_{i}}$ is increased above a threshold value, $s_{c}$, the stationary solution bifurcates away from equipartition: an increasingly large fraction of the total energy is channelled into the lowest-frequency mode $\omega_{0}$, giving rise to classical phonon condensation. Numerical integration of the dimensionless form of Equation (14) confirms that this threshold sharpens with an increasing number of modes, *N*, approaching a sharp bifurcation in the thermodynamic limit [10].

Introducing $\tau =t\omega_{0}$, $y_{\omega_{i}}=\omega_{i}\langle J_{\omega_{i}}\rangle /\left(k_{B}T_{B}\right)$, $\alpha_{\omega_{i}}=\omega_{i}/\omega_{0}$, where $\omega_{0}=\min_{\omega }\omega$, the rate equations take the dimensionless form

(15) $${\dot{y}}_{\omega_{i}}=S_{\omega_{i}}+B_{\omega_{i}}\left(1-y_{\omega_{i}}\right)+\sum_{\omega_{j}\neq \omega_{i}}C_{\omega_{i}\omega_{j}}\left[\frac{\alpha_{\omega_{i}}}{\alpha_{\omega_{j}}}y_{\omega_{j}}-y_{\omega_{i}}+\frac{\alpha_{\omega_{j}}-\alpha_{\omega_{i}}}{\alpha_{\omega_{j}}}y_{\omega_{i}}y_{\omega_{j}}\right]+\left(\mathrm{quartic}\right),$$

with a dimensionless control parameter, $S=S_{\omega_{i}}$ (assumed to be mode-independent). The equilibrium solution is uniformly $y_{\omega_{i}}=1$; condensation corresponds to a stationary solution with $y_{\omega_{0}}\gg 1$ and $y_{\omega_{i}}\ll y_{\omega_{0}}$ for $\omega_{i}>\omega_{0}$.

## 4. Classical Phonon Condensation

### 4.1. Stationary States and Order Parameters

The stationary solutions (${\dot{y}}_{\omega_{i}}=0$) at a small *S* are the equipartition fixed point, $y_{\omega_{i}}=1$, invariant under permutations of mode labels. Above a certain threshold, $S_{c}$, the nonlinear coupling breaks this permutation symmetry: energy flows preferentially toward $\omega_{0}$.

Two order parameters quantify this. The condensation index [22]

(16) $$E_{y}=\frac{y_{\omega_{0}}/{\tilde{y}}_{\omega_{0}}}{\sum_{i=0}^{N}y_{\omega_{i}}},\;{\tilde{y}}_{\omega_{0}}=1+S/B,$$

takes $E_{y}=0$ at equipartition and $E_{y}=1$ at full condensation. The ratio $p_{1}/p_{0}$ (where $p_{i}=y_{\omega_{i}}/\sum_{j}y_{\omega_{j}}$) goes from 1 to 0 at the same transition.

### 4.2. Numerical Evidence

The rate equations are numerically integrated for N+1 equally spaced modes, $\omega_{n}=\omega_{0}\left(1+n/N\right)$, starting from $y_{\omega_{i}}\left(0\right)=1$. Parameters: B=1, C=0.1, quartic coupling $\Upsilon^{\left(1\right)}=\Upsilon^{\left(2\right)}=10^{-4}$ [10].

**Figure 1:**
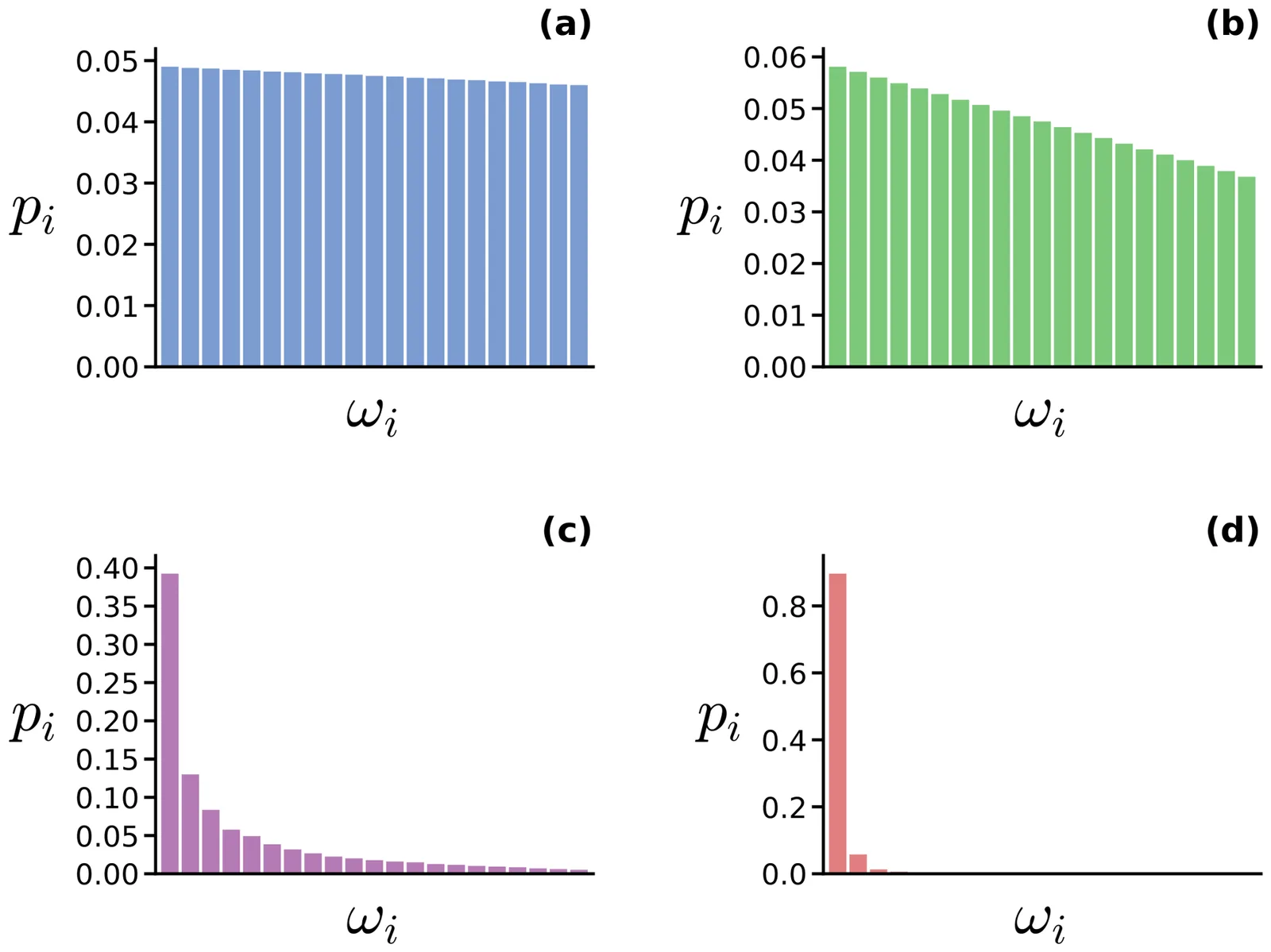
Classical Fröhlich-like condensation. Normalized energy fractions, $p_{i}$, in the normal modes vs. mode frequencies, $\omega_{i}$, for N=20 modes. As the energy input rate, *S*, increases, deviations from equipartition grow progressively. Panels (**a**–**d**) correspond to S=0.1 (blue), 1 (green), 10 (purple), and 100 (pink). Equipartition gives equal bar heights; note the increasing concentration of energy in the lowest-frequency mode at large *S*. From Ref. [10].

**Figure 2:**
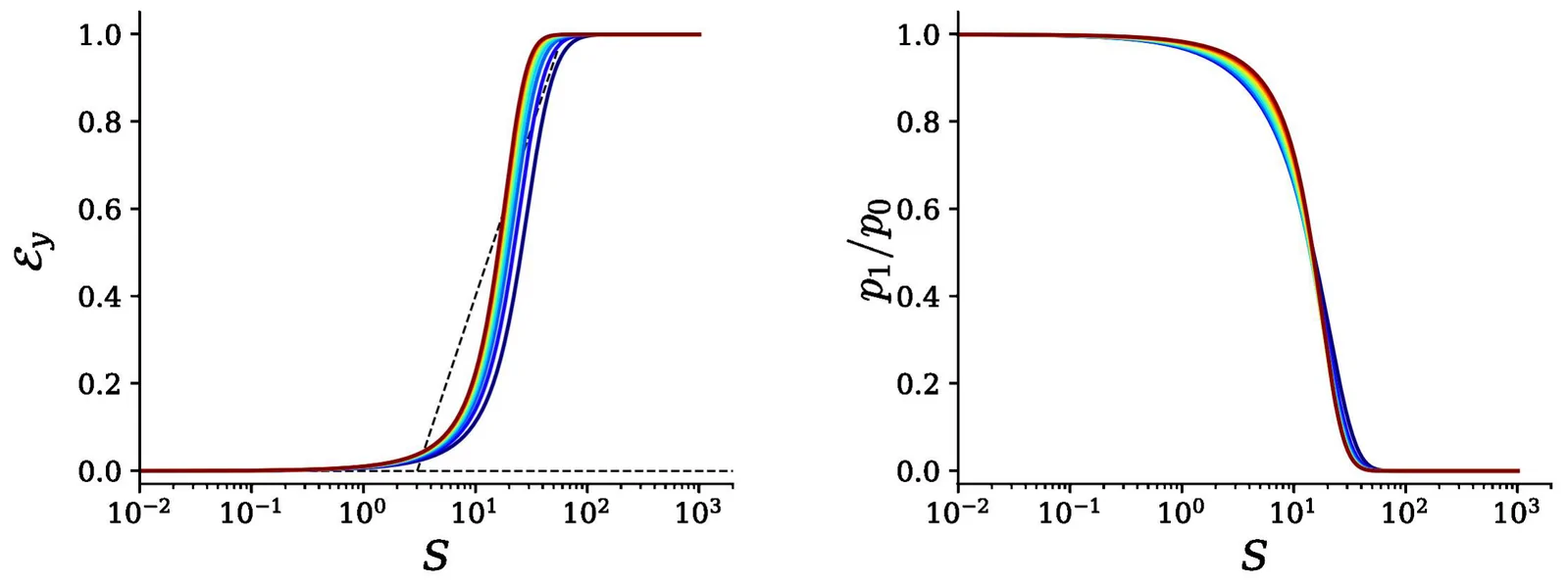
Classical Fröhlich-like condensation. Condensation index, $E_{y}$ ((**left**) panel), and ratio, $p_{1}/p_{0}$ ((**right**) panel), vs. energy input rate, *S*, for increasing mode number, $N_{\mathrm{sys}}$: from right to left, the curves are N=11 (black), 21 (blue), 41 (light blue), 101 (green), 201 (orange), 301 (brown). At equipartition, $E_{y}=0$, $p_{1}/p_{0}=1$; at full condensation, $E_{y}=1$, $p_{1}/p_{0}=0$. The dashed oblique line marks the inflection tangent as a guide to a possible asymptotic bifurcation. From Ref. [10].

As *S* increases, $p_{0}$ grows, while all $p_{i}$ (i>0) decrease. The condensation index $E_{y}\left(S\right)$ and the ratio $\left(p_{1}/p_{0}\right)\left(S\right)$ display a crossover near S≈10 that sharpens progressively with *N*, with curves for different *N* values crossing at a common point whose slope grows with *N*, suggestive of a sharp bifurcation in the large-*N* limit (see Figure 1 and Figure 2). This finite-size sharpening mirrors the behaviour of order–parameter curves near second-order equilibrium phase transitions.

The dynamics reveals a hierarchy: the highest-frequency modes lose energy first at increasing *S*; lower modes follow; eventually, all modes deplete except $\omega_{0}$, which absorbs the surplus and saturates at $p_{0}\to 1$. The saturation is robust and mode-number-independent at high drive.

### 4.3. Physical Interpretation

Classical phonon condensation is an instance of a nonequilibrium phase transition [23]: an open system with nonlinear internal couplings, dissipation, and external drive self-organises into a macroscopically ordered state when the energy-input rate exceeds a critical value. The genericity of this mechanism, depending only on nonlinear mode coupling, a thermal bath, and a source, not on molecular details, suggests the phenomenon should occur in real macromolecules. In the condensed state, the lowest-frequency mode carries a coherent oscillation of the entire molecule, activating a giant oscillating dipole moment that is a necessary prerequisite for long-range electrodynamic forces [11].

## 5. Long-Range Resonant Electrodynamic Interactions

### 5.1. Classical Electrodynamic Hamiltonian for Two Dipoles

The electrodynamic interaction between two coherently oscillating macromolecules is modelled at the classical level as a pair of driven harmonic oscillators coupled through the retarded electromagnetic field of the intervening medium. Each macromolecule is represented by its dominant polar mode, an oscillating electric dipole, $\mu_{A,B}\left(t\right)$, at a natural frequency, $\omega_{A,B}$, with an effective charge-to-mass ratio of $\zeta_{A,B}=Q_{A,B}^{2}/m_{A,B}$, where $Q_{A,B}$ and $m_{A,B}$ are the effective charge and mass of each dipole. This representation is appropriate because the low-frequency collective vibrations of macromolecules (ω/2π∼0.1–1 THz) fall well within the classical regime, $k_{B}T/\hbar \omega \gg 1$, at room temperature, and the dominant electrodynamic coupling involves the dipole moment of each molecule rather than higher multipoles.

The coupling is mediated by the electric field where each dipole radiates into the surrounding medium, here described by the frequency-dependent permittivity, ε(ω). The Debye screening that suppresses static electrostatic forces in the ionic cellular environment becomes ineffective at frequencies above ∼250 MHz [11], so that the oscillating dipole fields can propagate without ionic screening at the sub-THz frequencies of interest. The aqueous, ionic cellular environment therefore plays a genuinely dual role in this channel: at the sub-THz frequencies of interest, ions can no longer track the field’s oscillation, so the static Debye screening that would otherwise mask the interaction breaks down, while ε(ω) itself, through the retarded phase, $\omega \sqrt{\varepsilon^{\prime }\left(\omega \right)}\;r/c$, in Equation (19), sets the field’s effective phase velocity between the two dipoles and, through its imaginary part, governs the dissipative damping of that field with distance. Water is thus neither a passive spectator nor a simple obstacle here, but an active part of the medium that determines both whether the long-range term survives at all and how it decays with distance. In the dipole approximation, the equations of motion are [11]

(17) $${\ddot{\mu }}_{A}+\omega_{A}^{2}\mu_{A}=\zeta_{A}E_{B}\left(r_{A},t\right),$$

(18) $${\ddot{\mu }}_{B}+\omega_{B}^{2}\mu_{B}=\zeta_{B}E_{A}\left(r_{B},t\right),$$

where $E_{A(B)}\left(r_{B(A)},t\right)$ is the electric field created by dipole *A* (resp. *B*) at the location of its partner. Damping terms, $\gamma_{A,B}{\dot{\mu }}_{A,B}$, and anharmonic contributions are present in the full equations of motion [11] but are omitted here as the focus is on the conservative normal-mode structure from which the interaction potential is derived.

The field, $E_{A(B)}$, at the position of the partner is linear in the source dipole and is fully characterised, for a given separation, *r*, and frequency, by the electric susceptibility tensor, $\chi_{ij}\left(r,\omega \right)$, of the medium, whose explicit form is obtained from the solution of the d’Alembert equation for the vector potential in the Lorenz gauge [11]. Taking the *z*-axis along the intermolecular separation, r, the tensor is diagonal with elements

(19) $$\chi_{11}^{\prime }\left(r,\omega \right)=\chi_{22}^{\prime }\left(r,\omega \right)=-\frac{\cos(\omega \sqrt{\varepsilon^{\prime }}r/c)}{\varepsilon^{\prime }\left(\omega \right)r^{3}}\left(1-\frac{\omega^{2}\varepsilon^{\prime }\left(\omega \right)r^{2}}{c^{2}}\right)+\ldots ,\;\chi_{33}^{\prime }\left(r,\omega \right)=-2\chi_{11}^{\prime }\left(r,\omega \right)/\left(1-\ldots \right),$$

where $\chi_{ii}^{\prime }$ denotes the real (in-phase) part of $\chi_{ii}$, which is the component that contributes to the conservative interaction potential. In the near zone (r≪c/ω), and for a non-dispersive background ($\varepsilon^{\prime }=\mathrm{const}$), Equation (19) reduces to the static dipole propagator, $\chi_{ii}^{\prime }∼\sigma_{i}/\left(\varepsilon^{\prime }r^{3}\right)$ (with $\sigma_{1,2}=-1$, $\sigma_{3}=2$), while at finite frequency, the factor ε(ω) renormalises this scaling and the retardation corrections introduce oscillatory $r^{-2}$ and $r^{-1}$ terms whose importance grows with *r*.

The normal frequencies, $\omega_{N}$, of the dissipation-free coupled system are obtained by substituting the Fourier ansatz $\mu_{A,B}\left(t\right)=\mu_{A,B}^{\left(0\right)}e^{-i\omega_{N}t}$ into Equations (17) and (18) and requiring non-trivial solutions. The resulting secular condition is

(20) $$\left(\omega_{A}^{2}-\omega_{N}^{2}\right)\left(\omega_{B}^{2}-\omega_{N}^{2}\right)=\zeta_{A}\zeta_{B}{\left[\chi_{ii}^{\prime }\left(r,\omega_{N}\right)\right]}^{2},$$

which is an implicit equation in $\omega_{N}$ because the right-hand side itself depends on $\omega_{N}$ through the medium response. This implicitness is not a minor technicality: as shown in Ref. [11], a naive explicit approximation of the right-hand side at zeroth order in the coupling leads to the erroneous conclusion, originally reached by Fröhlich, that long-range resonant interactions of the form $U∼1/r^{3}$ survive at thermal equilibrium. The exact treatment via suitable integration shows that these contributions cancel identically at thermal equilibrium, reducing *U* to a short-range $1/r^{6}$ potential.

The solutions of Equation (20) are obtained analytically by a contour-integral method in the complex plane: the Lagrange inversion theorem, combined with Rouché’s theorem, allows one to resolve the implicit $\omega_{N}$-dependence of the right-hand side and yields the normal-mode frequencies as controlled perturbative expansions in the coupling $\zeta_{A}\zeta_{B}{\left[\chi_{ii}^{\prime }\right]}^{2}$, uniformly valid across the resonant ($\omega_{A}≃\omega_{B}$) and off-resonant ($\omega_{A}\gg \omega_{B}$) regimes. The key result is that the two regimes differ qualitatively in the range of the resulting interaction: the off-resonance the potential scales as $1/r^{6}$ (short-range, van der Waals-like), whereas, at resonance, it scales as $1/r^{3}$ in the near zone and as 1/r in the far zone, and is long-range at all distances. It is this selectivity of the resonant interaction that makes it a candidate mechanism for specific biomolecular recognition.

### 5.2. Off-Resonance and Resonant Interaction Potentials

The normal-mode frequencies obtained from Equation (20) determine the interaction energy through the shift they impart to the bare frequencies, $\omega_{A,B}$. The qualitative character of this shift depends critically on whether the two dipoles are in resonance or not.

#### 5.2.1. Off-Resonance Regime

When $\omega_{A}\gg \omega_{B}$, the right-hand side of Equation (20) is small compared with the unperturbed product, ${\left(\omega_{A}^{2}-\omega_{B}^{2}\right)}^{2}$, and the secular equation can be solved perturbatively. In the leading order, the frequency shift of each mode is proportional to $\zeta_{A}\zeta_{B}{\left[\chi_{ii}^{\prime }\right]}^{2}/\left(\omega_{A}^{2}-\omega_{B}^{2}\right)$, i.e., it is *second-order* in the coupling $\chi_{ii}^{\prime }$. Since $\chi_{ii}^{\prime }∼r^{-3}$ is in the near zone, the resulting interaction potential scales as $U\left(r\right)\propto r^{-6}$, which is a short-range, van der Waals-like potential, entirely analogous to the London dispersion interaction between non-resonant fluctuating dipoles.

#### 5.2.2. Resonant Regime

At resonance ($\omega_{A}\approx \omega_{B}=\omega_{0}$), the structure of Equation (20) changes qualitatively. Setting $\omega_{N}=\omega_{0}+\delta \omega$ and expanding to first order in δω, the left-hand side becomes ${\left(\omega_{0}^{2}-\omega_{N}^{2}\right)}^{2}\approx 4\omega_{0}^{2}{\left(\delta \omega \right)}^{2}$, so the secular equation reduces to a perfect square and yields

(21) $$\omega_{i,\pm }\left(r\right)≃\omega_{0}\pm \sqrt{\zeta_{A}\zeta_{B}}\;\frac{\chi_{ii}^{\prime }\left(r,\omega_{0}\right)}{2\omega_{0}}.$$

The frequency shift is now *first-order* in $\chi_{ii}^{\prime }$, not quadratic. This is the key distinction from the off-resonance case: the square-root structure of the resonant secular equation lifts the second-order suppression and produces a qualitatively stronger coupling.

The total interaction energy is expressed in terms of the adiabatic action variables, $J_{i,\pm }=E_{i,\pm }/\omega_{i,\pm }$, of the two normal modes, where $E_{i,\pm }$ is the energy stored in the ± branch of the polarisation component, *i*. Summing over the three spatial components,

(22) $$U\left(r\right)=\sum_{i=1}^{3}\Delta \omega_{0,i}\left(r\right)\;\left(J_{i,+}-J_{i,-}\right).$$

In the near zone ($r\ll c/\omega_{0}$), the susceptibility scales as $\chi_{ii}^{\prime }∼r^{-3}$, so $\Delta \omega_{0,i}∼r^{-3}$ and

(23) $$U\left(r\right)\;∼\;\pm \frac{1}{r^{3}},$$

which is a genuinely *long-range* interaction: the exponent drops from −6 in the off-resonance case to −3 at resonance, reflecting the fact that *U* is now linear rather than quadratic in terms of susceptibility, $\chi_{ii}^{\prime }$. The sign and magnitude are controlled by the action difference, $J_{i,+}-J_{i,-}$: at thermal equilibrium, this difference vanishes at first order, and the long-range term disappears, recovering a $1/r^{6}$ free energy. Out of equilibrium, when phonon condensation concentrates energy in one normal mode, $J_{i,+}\gg J_{i,-}$, and the $1/r^{3}$ interaction is activated. The explicit behaviour of $\chi_{ii}^{\prime }\left(r,\omega \right)$ as a function of *r* for representative frequencies in the sub-THz and optical regimes is shown in Figure 1 of Ref. [11].

A crucial result is that resonant long-range interactions *vanish* at thermal equilibrium. At equilibrium, the Boltzmann distribution weights the two normal-mode energies equally, causing the action difference, $J_{i,+}-J_{i,-}$, in Equation (22) to vanish at first order, giving a short-range $1/r^{6}$ free energy [11]. Long-range resonant electrodynamic forces therefore require an out-of-equilibrium system, where energy concentration in one normal mode, precisely the condition delivered by phonon condensation, makes $J_{i,+}\gg J_{i,-}$.

## 6. Position-Space Classical Hamiltonian and Molecular Dynamics

The action-angle Hamiltonian of Section 3 is natural for perturbative rate equations but remote from the (q,p) variables used in the molecular dynamics force fields. A complementary classical Hamiltonian was formulated directly in the position space [12], opening a path toward connecting Fröhlich condensation to atomistic MD. This has motivated the study of the following model.

### The Position-Space Hamiltonian

**Figure 3:**
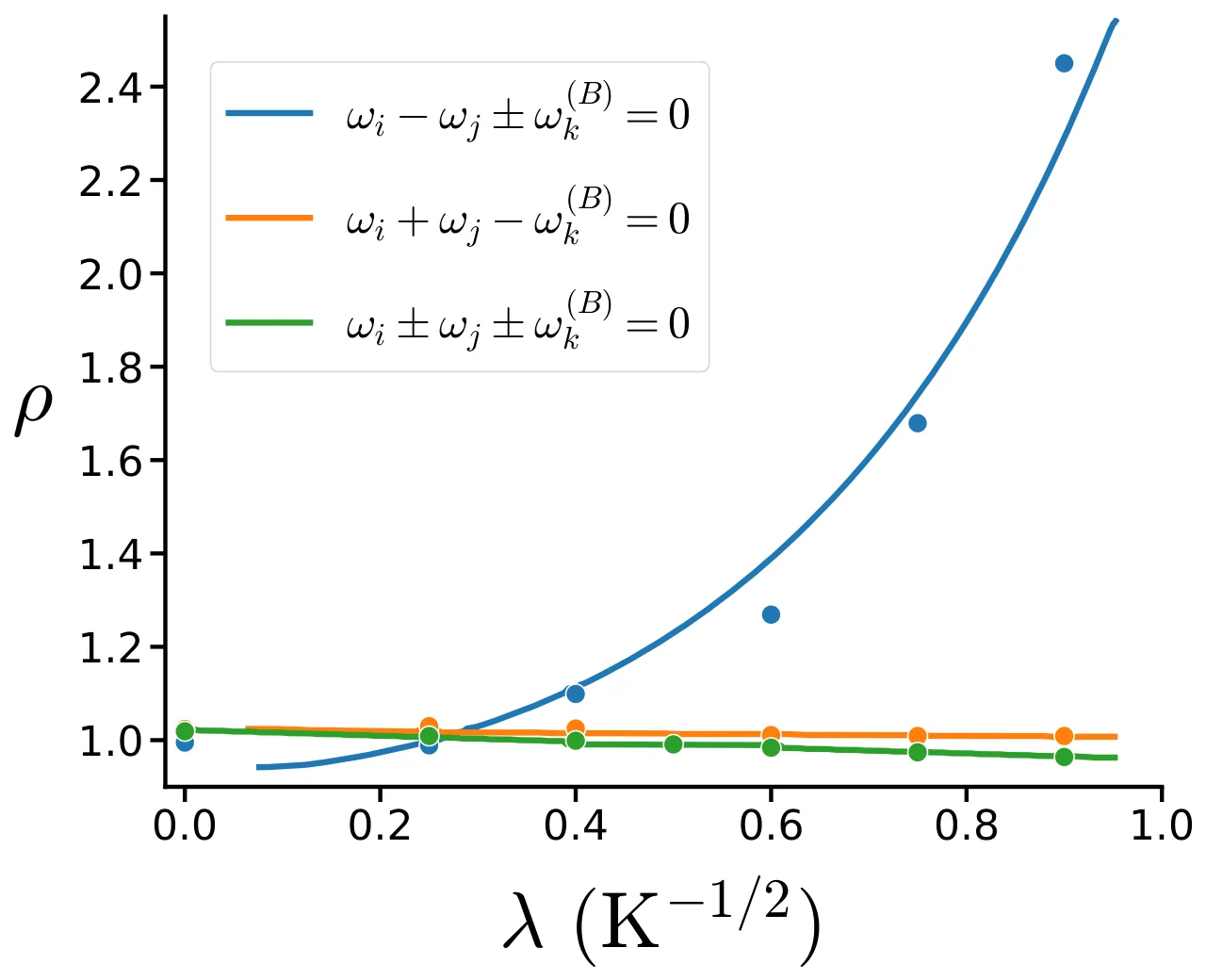
Condensation index ρ of a Fröhlich system from Hamiltonian dynamics, for coupling coefficients, $\lambda_{ijk}$, under three resonance conditions: $\omega_{i}-\omega_{j}\pm \omega_{k}^{\left(B\right)}=0$ (Fröhlich), $\omega_{i}+\omega_{j}-\omega_{k}^{\left(B\right)}=0$ (Lifshits), and $\omega_{i}\pm \omega_{j}\pm \omega_{k}^{\left(B\right)}=0$ (combined). From Ref. [12].

**Figure 4:**
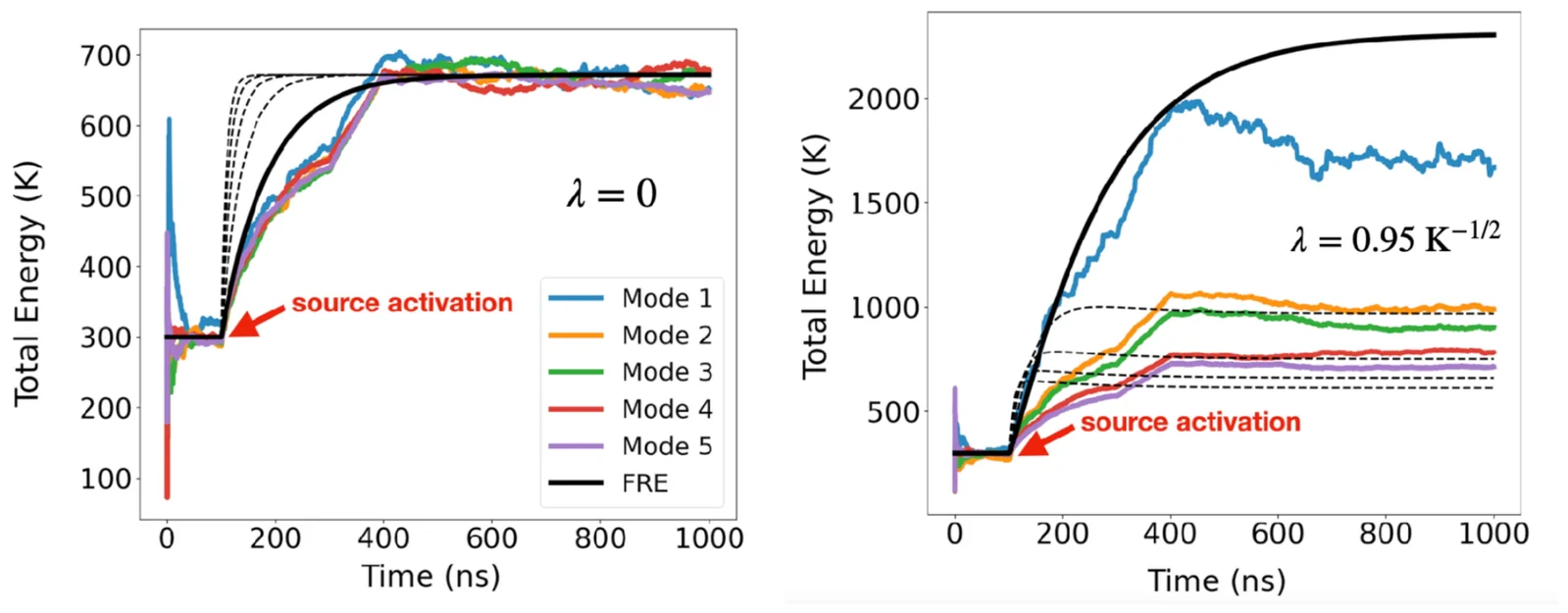
Time evolution of total energies (kinetic + potential) in a Fröhlich system of nine protein modes (first five shown), computed as 300 ns moving averages from Hamiltonian dynamics. Parameters: bath, T=300 K; source, $T_{S}=3000$ K; protein frequencies, $\omega_{1}=0.2$ THz to $\omega_{9}=1$ THz in 0.1 THz steps, $\phi_{ik}$, $\xi_{il}$, $\lambda_{ijk}$ from Equation (5) of Ref. [12] with ϕ=1.0 and ξ=0.4 (from 100 ns) unit masses; thermostat friction, 0.1 ps^−1^. Black curves: Fröhlich rate equations predictions from Equation (14) with α=0.02 ps (solid: mode 1; dashed: higher modes). (**Left**) panel: λ = 0; (**right**) panel: λ = 0.95 K^−1/2^. From Ref. [12].

The Hamiltonian and simulations reported in this subsection are those of Ref. [12]; we reproduce them here to connect the action-angle formulation of Section 3 to a form directly comparable with standard molecular dynamics force fields. The system Hamiltonian is $H=H_{0}+H_{\mathrm{int}}$, with a free part,

(24) $$H_{0}=\sum_{i=1}^{N}\left(\frac{p_{i}^{2}}{2m_{i}}+\frac{1}{2}m_{i}\omega_{i}^{2}q_{i}^{2}\right)+\sum_{k=1}^{N_{B}}\left(\frac{p_{k}^{(B)2}}{2m_{k}^{\left(B\right)}}+\frac{1}{2}m_{k}^{\left(B\right)}\omega_{k}^{(B)2}q_{k}^{(B)2}\right)+\sum_{l=1}^{N_{S}}\left(\frac{p_{l}^{(S)2}}{2m_{l}^{\left(S\right)}}+\frac{1}{2}m_{l}^{\left(S\right)}\omega_{l}^{(S)2}q_{l}^{(S)2}\right),$$

and interaction

(25) $$H_{\mathrm{int}}=\sum_{ik}\phi_{ik}\;q_{i}q_{k}^{\left(B\right)}+\sum_{il}\xi_{il}\;q_{i}q_{l}^{\left(S\right)}+\sum_{ijk}\lambda_{ijk}\;q_{i}q_{j}q_{k}^{\left(B\right)},$$

where $\phi_{ik}$, $\xi_{il}$, and $\lambda_{ijk}$ are linear protein–bath, linear protein–source, and nonlinear protein–protein–bath couplings. Second-order perturbation theory recovers the Fröhlich rate constants $\Phi_{i}$, $\Xi_{i}$, and $\Lambda_{ij}$, all depending on the resonance conditions [12]. Hamiltonian equations are integrated numerically with bath oscillators held at T=300 K by a Langevin thermostat [12]. The key findings are as follows: *(i)* Pure Fröhlich resonances ($\omega_{i}-\omega_{j}\pm \omega_{k}^{\left(B\right)}=0$) produce robust condensation (Figure 3); Lifshits sum resonances ($\omega_{i}+\omega_{j}-\omega_{k}^{\left(B\right)}=0$) destroy it. *(ii)* Replacing the bath-mediated trilinear coupling, $\lambda_{ijk}q_{i}q_{j}q_{k}^{\left(B\right)}$, by a protein-only coupling, $\lambda_{ijk}q_{i}q_{j}q_{k}$, completely suppresses condensation: bath mediation is physically essential, not a technical device. *(iii)* Strong condensates are found at T=300 K, contradicting an earlier study [22] that used constant coupling coefficients (Figure 4). The discrepancy traces to the coupling parametrisation: coefficients proportional to $\sqrt{m_{i}m_{k}^{\left(B\right)}\omega_{i}\omega_{k}^{\left(B\right)}}$ ensure all modes contribute equally to the interaction energy at equipartition.

## 7. Electron–Phonon Hamiltonian and DNA–Protein Co-Resonance

### 7.1. Motivation and Biological Context

Beyond collective vibrational (phonon) degrees of freedom, the electronic degrees of freedom of biomolecules provide a further and conceptually distinct channel through which selective long-range electrodynamic interactions can be generated [13]. This possibility is motivated by three converging lines of evidence. First, the Resonant Recognition Model (RRM) [24,25] showed empirically that a Fourier analysis of the electron–ion interaction potentials (EIIPs) along a protein or DNA sequence produces spectral peaks that correlate with biological activity. Second, electron transport along DNA and along protein backbones is an experimentally confirmed phenomenon [26,27]. We note that the range, mechanism, and efficiency of DNA-mediated charge transport have themselves been the subject of a long-running debate, from the early “insulator or wire” controversy [28] to subsequent mechanistic refinements distinguishing coherent tunnelling from thermally activated hopping over different distance regimes [29]. The electron–phonon model adopted in Ref. [13] does not require long-range coherent conduction per se: it only requires that local, sequence-dependent electron–phonon coupling along a short recognition window produces a sequence-specific current signature, a considerably weaker and less contested requirement than genuine long-distance DNA conductivity. Third, a previous study [14] established that the electron current flowing along a DNA fragment under an external energy supply can exhibit either a broad or a sharply peaked frequency spectrum depending on the excitation site and energy, suggesting that co-resonance between DNA and enzyme currents could provide a sequence-specific electrodynamic signal.

### 7.2. The Davydov–Holstein–Fröhlich Hamiltonian

To model electron–phonon motion along a biomolecular chain of *N* sites (nucleotides or amino acids), the following Hamiltonian is adopted [13]:

(26) $$\hat{H}={\hat{H}}_{\mathrm{el}}+{\hat{H}}_{\mathrm{ph}}+{\hat{H}}_{\mathrm{int}},$$

with an electronic part

(27) $${\hat{H}}_{\mathrm{el}}=\sum_{n=1}^{N}\left[E_{0}{\hat{B}}_{n}^{†}{\hat{B}}_{n}+\epsilon \langle {\hat{B}}_{n}^{†}{\hat{B}}_{n}\rangle {\hat{B}}_{n}^{†}{\hat{B}}_{n}+J_{n}\left({\hat{B}}_{n}^{†}{\hat{B}}_{n+1}+{\hat{B}}_{n}^{†}{\hat{B}}_{n-1}\right)\right],$$

a phononic part (with an anharmonic term)

(28) $${\hat{H}}_{\mathrm{ph}}=\frac{1}{2}\sum_{n}\left[\frac{{\hat{p}}_{n}^{2}}{M_{n}}+\Omega_{n}{\left({\hat{u}}_{n+1}-{\hat{u}}_{n}\right)}^{2}+\frac{1}{2}\mu {\left({\hat{u}}_{n+1}-{\hat{u}}_{n}\right)}^{4}\right],$$

and an electron–phonon interaction

(29) $${\hat{H}}_{\mathrm{int}}=\sum_{n}\chi_{n}\left({\hat{u}}_{n+1}-{\hat{u}}_{n}\right){\hat{B}}_{n}^{†}{\hat{B}}_{n}.$$

Here, ${\hat{B}}_{n}$ and ${\hat{B}}_{n}^{†}$ are the lowering and raising operators at lattice site *n*; $E_{0}$ is the initial electron excitation energy; ϵ is the nonlinear electron self-trapping constant; $J_{n}$ is the site-dependent electron tunnelling amplitude; ${\hat{u}}_{n}$ and ${\hat{p}}_{n}$ are the longitudinal displacement and momentum of the *n*-th nucleotide or amino acid, according to the kind of molecule, DNA or protein, respectively; $\Omega_{n}$ is the site-dependent spring constant; $M_{n}$ is the site mass; μ is the particle–particle (nucleotide or amino acid) anharmonic coupling; and $\chi_{n}$ is the site-dependent electron–phonon coupling. The coupling parameters $J_{n}$ and $\chi_{n}$ are site-dependent, encoding the specific sequence of nucleotides (for DNA) or amino acids (for protein).

### 7.3. TDVP Applied to the Davydov Ansatz

The TDVP is again the instrument of choice. The trial wave function is written in the Davydov factorised form

(30) |ψ(t)〉=|Ψ(t)〉|Φ(t)〉,

where the electronic part |Ψ(t)〉 is a single-excitation state

(31) $$|\Psi \left(t\right)\rangle =\sum_{n}C_{n}\left(t\right){\hat{B}}_{n}^{†}{|0\rangle }_{\mathrm{el}},$$

with probability amplitudes, $C_{n}\left(t\right)$, and the phononic part, |Φ(t)〉, is a displaced coherent state

(32) $$|\Phi \left(t\right)\rangle =e^{-\frac{i}{\hbar }\sum_{n}\left[\beta_{n}\left(t\right){\hat{p}}_{n}-\pi_{n}\left(t\right){\hat{u}}_{n}\right]}{|0\rangle }_{\mathrm{ph}},$$

so that $\langle \Phi |{\hat{u}}_{n}|\Phi \rangle =\beta_{n}\left(t\right)$ and $\langle \Phi |{\hat{p}}_{n}|\Phi \rangle =\pi_{n}\left(t\right)$.

Applying the stationary-action condition, δS=0, with the Lagrangian $L\left(t\right)=i\hbar \langle \psi |\partial_{t}|\psi \rangle -\langle \psi |\hat{H}|\psi \rangle$ yields equations of motion for the variational parameters $\left(C_{n},C_{n}^{*},\beta_{n},\pi_{n}\right)$:

(33) $$i\hbar {\dot{C}}_{n}=\partial_{C_{n}^{*}}H,\;{\dot{\beta }}_{n}=\partial_{\pi_{n}}H,\;{\dot{\pi }}_{n}=-\partial_{\beta_{n}}H,$$

where $H=\langle \psi |\hat{H}|\psi \rangle$ is the classical Hamiltonian obtained as the expectation value.

Evaluating *H* explicitly [13]:

(34) $$\begin{array}{cc} H & =\sum_{n}[E_{0}|C_{n}{|}^{2}+\epsilon {\left|C_{n}\right|}^{4}+J_{n}\left(C_{n}^{*}C_{n+1}+C_{n+1}^{*}C_{n}\right)\\ \; & \;\;+\frac{1}{2}\;\left(\frac{\pi_{n}^{2}}{M_{n}}+\Omega_{n}{\left(\beta_{n+1}-\beta_{n}\right)}^{2}+\frac{\mu }{2}{\left(\beta_{n+1}-\beta_{n}\right)}^{4}\right)\\ \; & \;\;+\chi_{n}\left(\beta_{n+1}-\beta_{n}\right){\left|C_{n}\right|}^{2}]. \end{array}$$

The equations of motion that follow from Equations (33) and (34) are formally classical but describe the time evolution of quantum expectation values:

(35) $$\begin{array}{cc} i\hbar {\dot{C}}_{n} & =\left[E_{0}+2\epsilon {\left|C_{n}\right|}^{2}+\chi_{n}\left(\beta_{n+1}-\beta_{n}\right)\right]C_{n}\\ \; & \;+J_{n}C_{n+1}+J_{n-1}C_{n-1}, \end{array}$$

(36) $$\begin{array}{cc} M_{n}{\ddot{\beta }}_{n} & =\Omega_{n}\beta_{n+1}+\Omega_{n-1}\beta_{n-1}-\Omega_{n-1}\beta_{n}-\Omega_{n}\beta_{n}\\ \; & \;+\chi_{n}|C_{n}{|}^{2}-\chi_{n-1}{\left|C_{n-1}\right|}^{2}\\ \; & \;+\mu \left[{\left(\beta_{n+1}-\beta_{n}\right)}^{3}-{\left(\beta_{n}-\beta_{n-1}\right)}^{3}\right]. \end{array}$$

Equations (35) and (36) couple the quantum probability amplitudes for electron propagation to the classical phonon displacements of the chain, encoding the full electron–phonon dynamics of the specific biomolecular sequence.

### 7.4. Physical Parameters and Numerical Strategy

In Ref. [13], the DNA–protein system studied is a 66 bp DNA oligonucleotide interacting with the EcoRI restriction enzyme (276 amino acids). The six base pairs (5′-CTTAAG-3′) refer specifically to the EcoRI recognition sequence embedded within this 66 bp oligomer, not to the size of the simulated chain: six is simply the length of the palindromic motif that this restriction enzyme recognises biologically, so it is dictated by the biology of the enzyme–substrate pair rather than chosen for computational convenience. The 66 bp length of the flanking DNA was chosen to be long enough that the recognition site sits away from the chain boundaries, avoiding finite-size artefacts in the electron current at the strand ends, while remaining computationally tractable for the Euler predictor–corrector integration described below. Longer DNA segments (hundreds of base pairs) and larger protein partners are, in principle, within reach of the same TDVP–Davydov formalism, at a computational cost that scales with the number of coupled electron–phonon sites; extending the model to genomic-scale sequences, or benchmarking it against curated variant databases such as MITOMAP, would allow a systematic test of whether the co-resonance signature tracks known conserved or disease-associated motifs, and is a natural next step for this programme, rather than a result available in the present review. The site-dependent parameters $J_{n}$ and $\chi_{n}$ are determined from the electron–ion interaction potentials (EIIPs) of each nucleotide (A, T, G, C) and each amino acid, tabulated in Refs. [24,25,30]. The tunnelling amplitudes are estimated from the transmission probability P(n→n±1) through the potential barrier between adjacent sites; the site-dependent electron–phonon coupling is estimated as $\chi_{n}=dE/dx=\left(E_{n+1}-E_{n}\right)/a$, where *a* is the nearest-neighbour distance between particles (a=3.4 Å for nucleotides of DNA, 4.5 Å for the amino acids of enzyme).

The particle displacements are initialised at thermal equilibrium at T=310 K:

(37) $$\langle |b_{n}{\left(0\right)|\rangle }_{n}=\sqrt{\frac{k_{B}T}{\hbar \omega \Omega^{\prime }}},\;\langle |\pi_{n}\left(0\right){|\rangle }_{n}=\sqrt{\frac{k_{B}T}{\hbar \omega }}.$$

The electron wavefunction is initialised as a localised sech pulse centred at site $n_{0}$. Integration uses a symplectic leapfrog scheme for the phonon variables combined with an Euler predictor–corrector for the electron amplitudes, with a relative energy conservation of $\Delta E/E∼10^{-6}$.

### 7.5. Cross-Spectral Co-Resonance and Sequence Specificity

The primary observable is the cross-Fourier spectrum of the electron current densities along the two chains. The average electron current along chain α (α=1 for DNA, α=2 for EcoRI) is

(38) $$i_{\alpha }\left(t\right)=\frac{e\hbar }{2N_{\alpha }a_{\alpha }m_{e}i}\sum_{j=1}^{N_{\alpha }}\left(\Psi_{\alpha }^{*}\left(x_{j},t\right)\;\frac{\Psi_{\alpha }\left(x_{j+1},t\right)-\Psi_{\alpha }\left(x_{j-1},t\right)}{2}-c.c.\right),$$

and the cross-spectrum ${\tilde{i}}_{1}^{*}\left(\nu \right){\tilde{i}}_{2}\left(\nu \right)$ is computed from their Fourier transforms.

The central finding in [13] is illustrated schematically as follows. When the EcoRI recognition site on the DNA is the canonical palindromic sequence $3^{\prime }$-CTTAA|G-$5^{\prime }$ (where | marks the cleavage site), the cross-spectrum displays a *sharp co-resonance peak* at ν≈20–29 THz (depending on initial conditions), in quantitative agreement with the RRM prediction [24].

**Figure 5:**
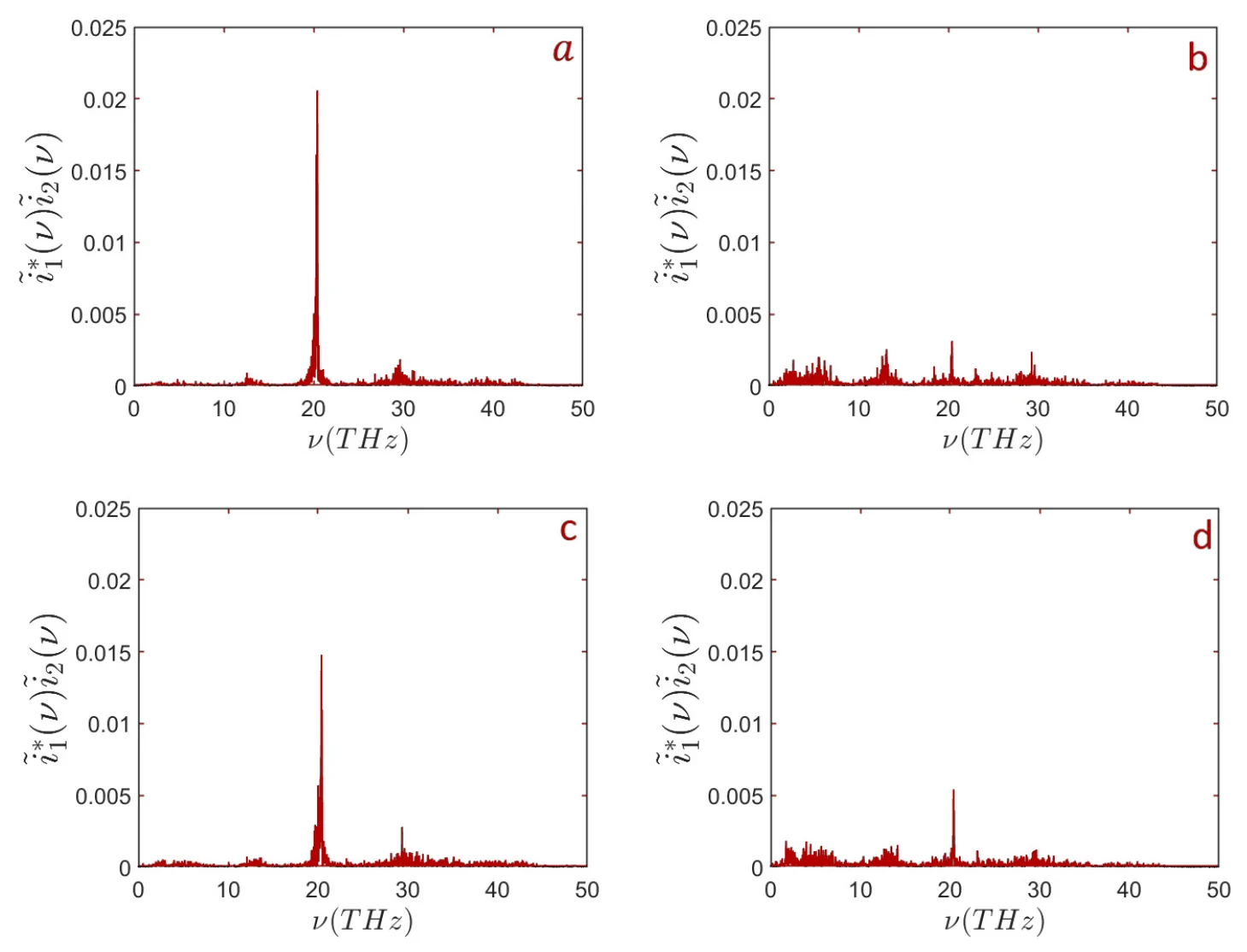
Cross frequency spectra between a DNA strand with $N_{1}=66$ nucleotides and the *Eco*RI enzyme with $N_{2}=276$ amino acids. Panels show DNA strand containing (**a**) the canonical target CTTAAG recognition site, (**b**) cyclic permutation of restriction site in (**a**) to AGCTTA, (**c**) one-nucleotide change from (**a**) with its base-pair complement to CATAAG, and (**d**) two-nucleotide change from (**a**) with their base-pair complements to GTTAAC. From Ref. [13].

**Figure 6:**
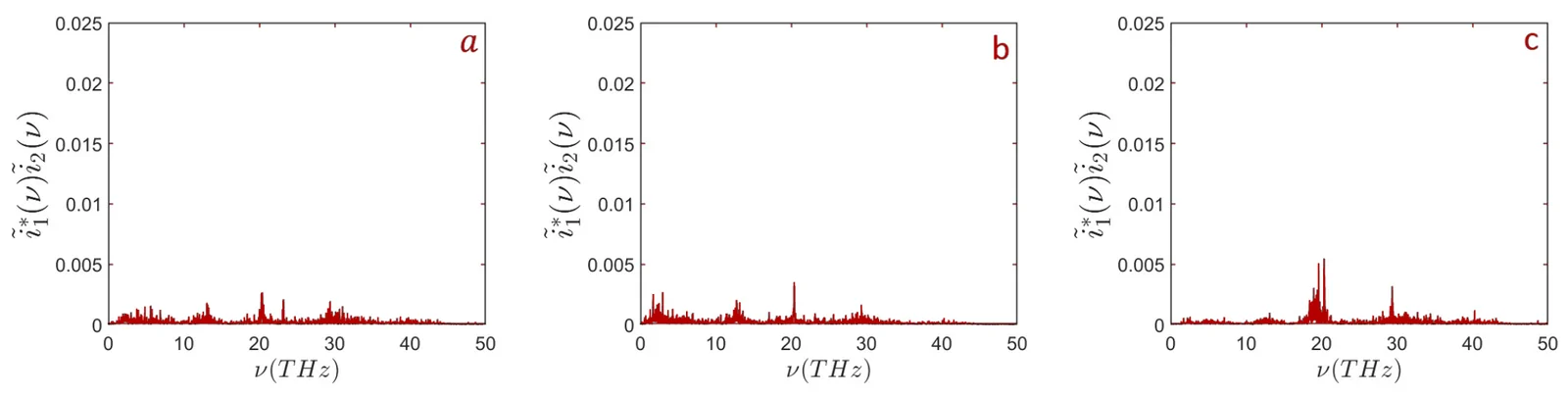
Cross-frequency spectra between a DNA strand and the *Eco*RI enzyme, under the same substrate and initial conditions as Figure 5. Panels show DNA strand containing: (**a**) randomized restriction site TCATGA; (**b**) one-nucleotide change from the canonical target CTTAAG to CTTAAC; (**c**) two-nucleotide change from the canonical target CTTAAG to CATATG. From Ref. [13].

When the recognition sequence is randomised (e.g., AGCTTA, TCATGA, AGATCT), the sharp peak disappears and is replaced by a broad, noisy spectrum (see Figure 5 and Figure 6). When one nucleotide of the recognition site is exchanged with its complement (e.g., CATAAG), the peak undergoes a modest broadening; two exchanges (e.g., GTTAAC) broaden it more. The co-resonance is therefore robustly and specifically associated with the canonical recognition sequence.

**Figure 7:**
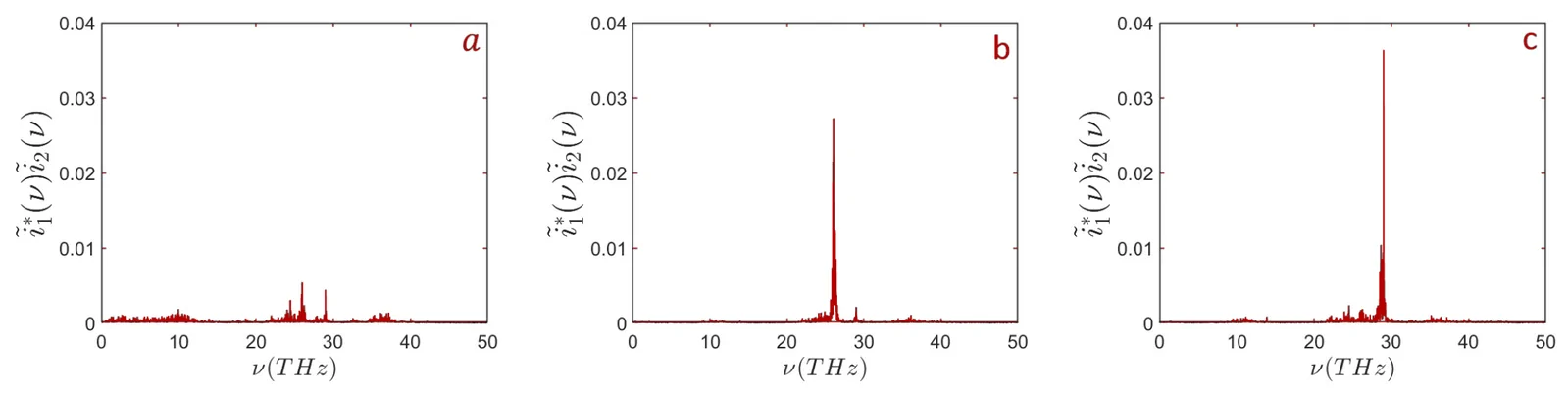
Cross-frequency spectra between a DNA strand and the *Eco*RI enzyme, under the same substrate and initial conditions as Figure 5. Panels show DNA strand containing (**a**) a randomized restriction site, AGATCT, (**b**) one-nucleotide change from the canonical target CTTAAG to CATAAG, and (**c**) two-nucleotide change from the canonical target CTTAAG to GTTATG. From Ref. [13].

**Figure 8:**
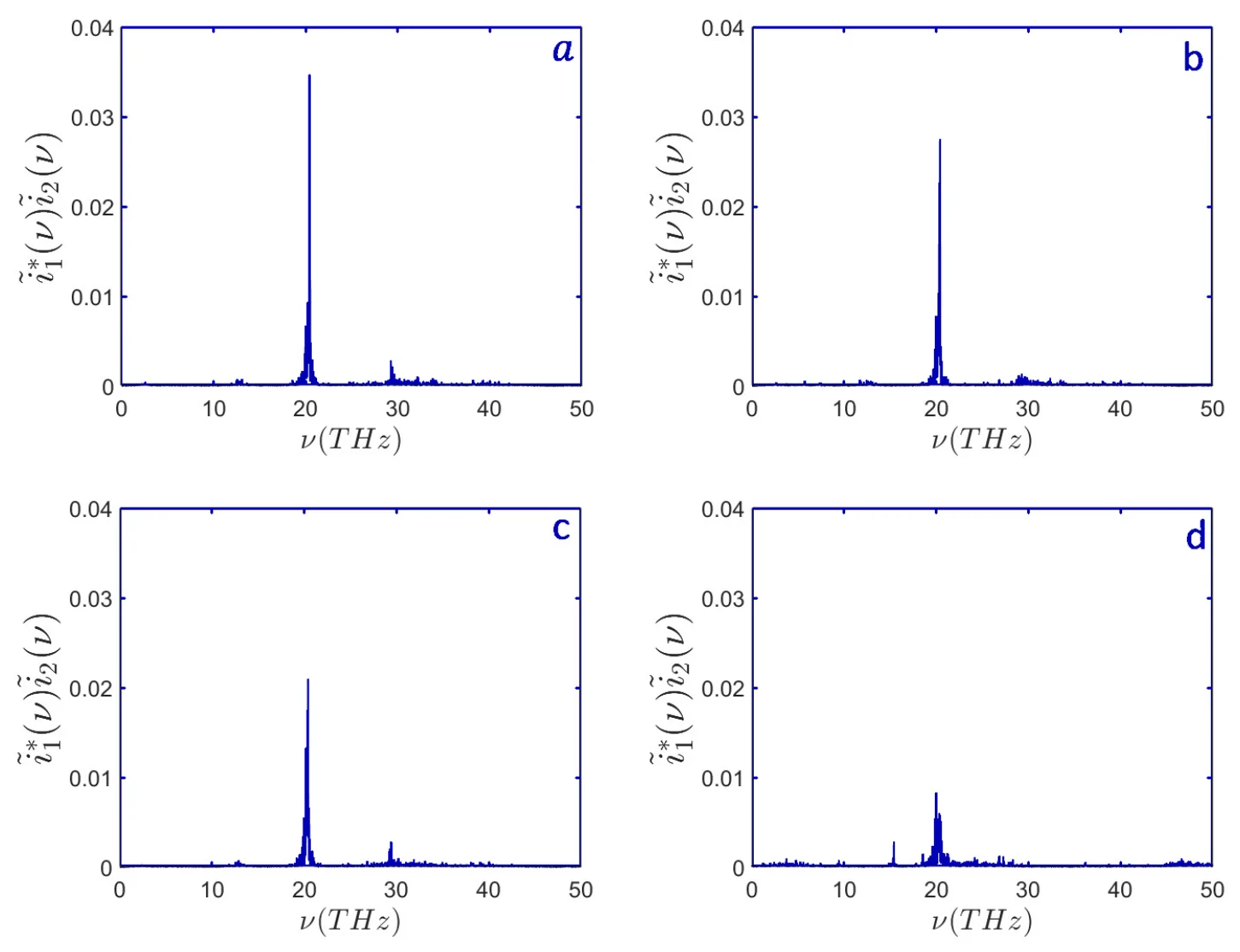
Cross-frequency spectra between the original substrate and the *Eco*RI mutants with the single-point mutations: (**a**) $Ala^{138}\to Thr$; (**b**) $Glu^{192}\to Lys$; (**c**) $His^{114}\to Tyr$; and (**d**) $Asp^{91}\to Asn$. The initial conditions are the same as in Figure 5. From Ref. [13].

The model also discriminates between point-mutation variants with known biological activity [13]: promiscuous mutations (Ala^138^ →Thr, Glu^192^ →Lys, His^114^ →Tyr) that allow binding to near-cognate sequences still yield a sharp co-resonance peak, while the catalytically inactivating mutation Asp^91^ →Asn produces a sizeable decrease in the peak (see Figure 7 and Figure 8). These findings are coherent with the experimental biochemical data.

### 7.6. Physical Interpretation and Comparison with the Vibrational Channel

The TDVP-derived equations of motion, (35) and (36), are formally classical but arise directly from a quantum Hamiltonian via the same variational dequantization procedure applied in Section 3 to the Wu–Austin model. The two applications of the TDVP operate on different physical degrees of freedom (collective vibrational modes vs. electron–phonon propagation) and at different energy scales (sub-THz vs. tens of THz), but share the same mathematical structure: a product ansatz over coherent (or single-excitation) states, a classical Lagrangian obtained as the expectation value of the Hamiltonian, and equations of motion for the classical parameters governing the trial state.

The co-resonance reported here constitutes a distinct and complementary mechanism for selective electrodynamic interactions between biomolecules. In the vibrational channel (Section 4 and Section 5), the relevant quantity is the oscillating dipole moment, carried by a collective phonon mode, and driven into a condensed state by external energy input. In the electronic channel (this section), the relevant quantity is the oscillating electron current density whose spectral overlap with that of the partner molecule determines the strength of the co-resonant coupling.

Both channels require out-of-equilibrium conditions for their activation: the phonon channel requires energy input exceeding the condensation threshold; the electronic channel requires electron excitation, provided in the cell, for example, by redox reactions, ATP-driven ion currents, or biophoton emission [13]. It is plausible that both channels are simultaneously active in vivo, and that they reinforce each other in determining the selectivity and range of biomolecular recognition.

The mediating role of the aqueous environment is highlighted by the Jaynes–Cummings-like interaction Hamiltonian [13] $H_{\mathrm{int}}=\hbar \sqrt{N}\;\gamma \left(a^{†}S^{-}+aS^{+}\right)$, where $a^{†}$ and *a* are the creation and annihilation operators for the DNA radiative electric field, $S^{\pm }$ are the raising/lowering operators for the collective orientational state of the *N* surrounding water dipoles, and the coupling scales as $\sqrt{N}$, suggesting that the large number of water molecules in a physiological domain provides a protective gap against thermalisation for the long-range correlations [13,31].

## 8. Experimental Evidence

### 8.1. Experimental Strategy

**Figure 9:**
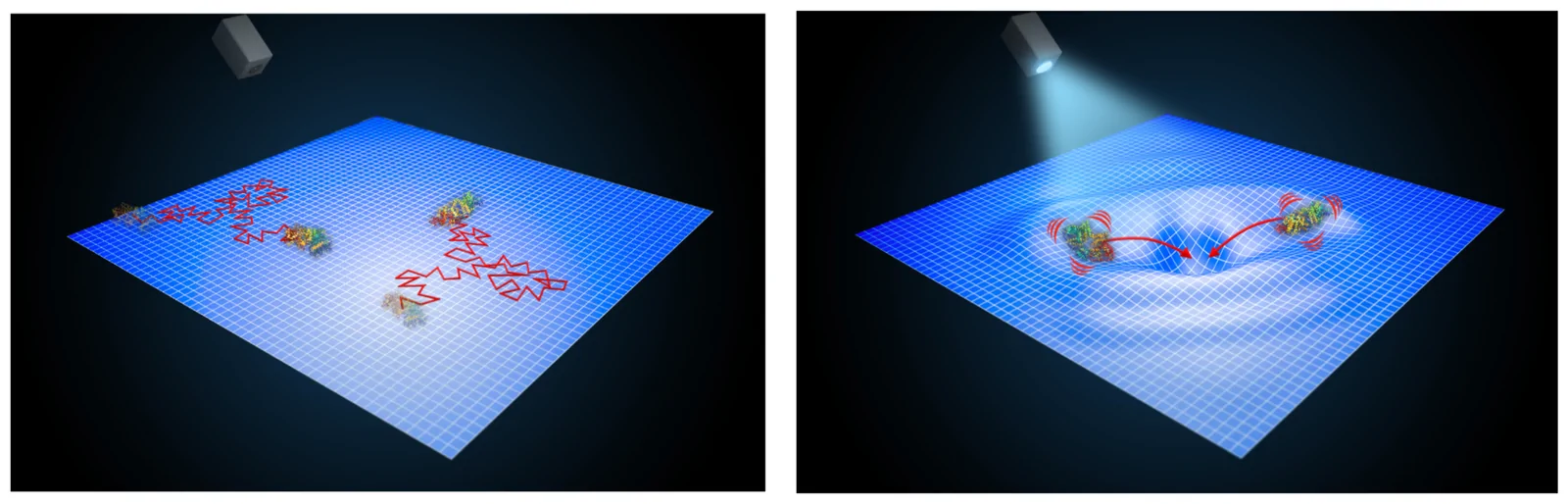
Long-range electrodynamic interactions—principle and experimental approaches.At thermal equilibrium, macromolecules show a Brownian diffusive motion in solution ((**left**) panel). By switching on an external energy source, molecules are in an out-of-thermal, equilibrium, collective vibrational state that can generate ED forces through associated large dipolar resonant oscillations ((**right**) panel). From Ref. [15].

All the experimental results presented in this section were obtained and first reported in Refs. [10,15]; the Wu–Austin/TDVP theory reviewed in Section 2, Section 3 and Section 4 and the electrodynamic force theory of Section 5 motivated the choice of observables described below, but no new experimental data are reported here. The unprecedented experimental programme outlined in the present section is focused on two observables predicted by theory. First, an out-of-equilibrium macromolecule undergoing phonon condensation should display a sharp sub-THz absorption feature that is absent at thermal equilibrium, with a threshold in the energy-input rate [10]. Second, if two such macromolecules interact via resonant electrodynamic forces, the collective oscillation frequency of each should shift as $\Delta \nu \propto 1/{\langle r\rangle }^{3}$ [11,15], and above a critical concentration, a clustering phase transition should occur [15]. (see Figure 9).

Out-of-equilibrium conditions are achieved by the optical pumping of fluorochrome-labelled proteins with a continuous-wave Argon laser (488 nm), inducing a “proteinquake” energy transfer to the vibrational modes. Two complementary THz near-field spectroscopy setups, a microwire probe and a plasmonic rectenna, operated in two independent laboratories, together with fluorescence correlation spectroscopy (FCS), serve as detectors.

### 8.2. Phonon Condensation in BSA

The first experimental test of the classical phonon condensation scenario was performed with bovine serum albumin (BSA), a globular protein of 67 kDa, chosen as a well-characterised model system [10]. Because BSA has no endogenous chromophore that is suitable for efficient optical pumping, five to six Alexa 488 fluorochrome molecules were covalently attached to its lysine residues and excited by an argon-ion laser at 488 nm. The energy difference between absorbed and re-emitted photons (≈0.19 eV per fluorochrome per incident photon) provides the energy input that drives the protein out of thermal equilibrium via the proteinquake mechanism [32]. Only a fraction of this non-radiative energy is expected to reach the collective low-frequency mode selectively: most of it is deposited locally near the fluorochrome and thermalises within picoseconds among the high-frequency, short-wavelength modes of the protein, exactly as in the proteinquake picture of Ref. [32]. It is the nonlinear, bath-mediated intermode coupling, χ, of Equation (1), not the direct funnelling of the absorbed photon energy, that subsequently channels a growing share of this locally thermalised energy into the lowest-frequency mode once the input rate exceeds threshold; the energy input rate, *W*, used in Equation (40) is therefore already the rate at which energy *enters this nonlinear redistribution process*, not the rate at which it is deposited directly into the condensing mode. A direct experimental measurement of the instantaneous partition between local heat and coherent collective motion is not yet available and is noted here as an open point.

**Figure 10:**
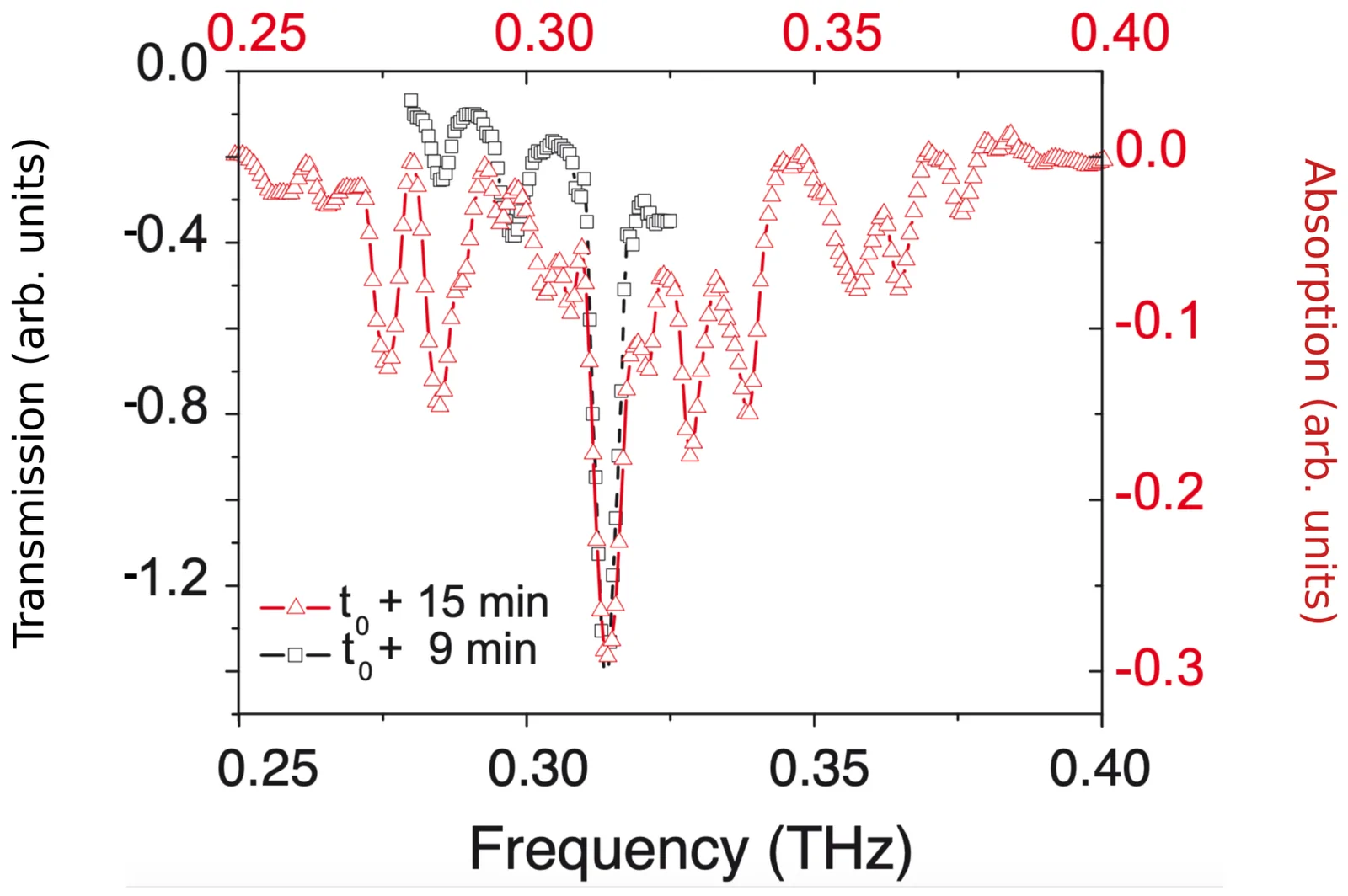
Differential transmission and absorption spectra as functions of the frequency. Comparison of the two normalized spectra for the longest illumination durations. From Ref. [10].

THz near-field absorption spectroscopy was performed simultaneously in two independent laboratories using complementary probe technologies: a 12 μm-diameter micro-coaxial wire (Montpellier) and a plasmonic bow-tie rectenna coupled to a plasma-wave FET (Rome). Both setups operate in the 0.22–0.33 THz range, with a spectral resolution below 300 Hz from continuous-wave Virginia Diodes sources. A principal resonance at ν=0.314 THz was reproducibly observed in both laboratories only when (i) Alexa 488 was bound to BSA and (ii) the laser was switched on; control measurements on the labelled BSA without laser illumination, on free dye without protein, and on the unlabelled BSA with illumination all showed no spectral features. (see Figure 10).

The resonance frequency is in quantitative agreement with the lowest spheroidal deformation mode of an elastic sphere,

(39) $$\nu_{0}=\frac{1}{2\pi }{\left[\frac{2(2l+1)(l-1)E}{\rho R_{H}^{2}}\right]}^{1/2},\;l=2,$$

giving $\nu_{0}\approx 0.308$ THz using the independently measured Young’s modulus at E=6.75 GPa and a hydrodynamic radius of $R_{H}=35$ Å [10].

A complementary, rough estimate treats BSA as a sphere split into two parts with masses of m=33 kDa joined by a spring with a stiffness of $k=EA_{0}/\ell_{0}$, giving ν≈0.300 THz [10], within 5% of the observed resonance, providing independent confirmation that the feature is a global deformation mode of the whole molecule. The two weaker resonances observed at 0.278 and 0.285 THz can be tentatively assigned to torsional modes at frequencies $\nu_{t}=\nu_{0}{\left[\left(2l+3\right)/\left(2\left(2l+1\right)\right)\right]}^{1/2}$ for l=2 and l=3, which yield 0.257 and 0.246 THz, respectively; the remaining discrepancy is consistent with the non-spherical shape of BSA, introducing different moments of inertia along different axes. The absorption line profile is well described by the Lorentzian expected for a damped harmonic oscillator (Equation (52) of Ref. [10]), with a quality factor of Q=Δν/ν≈50.

**Figure 11:**
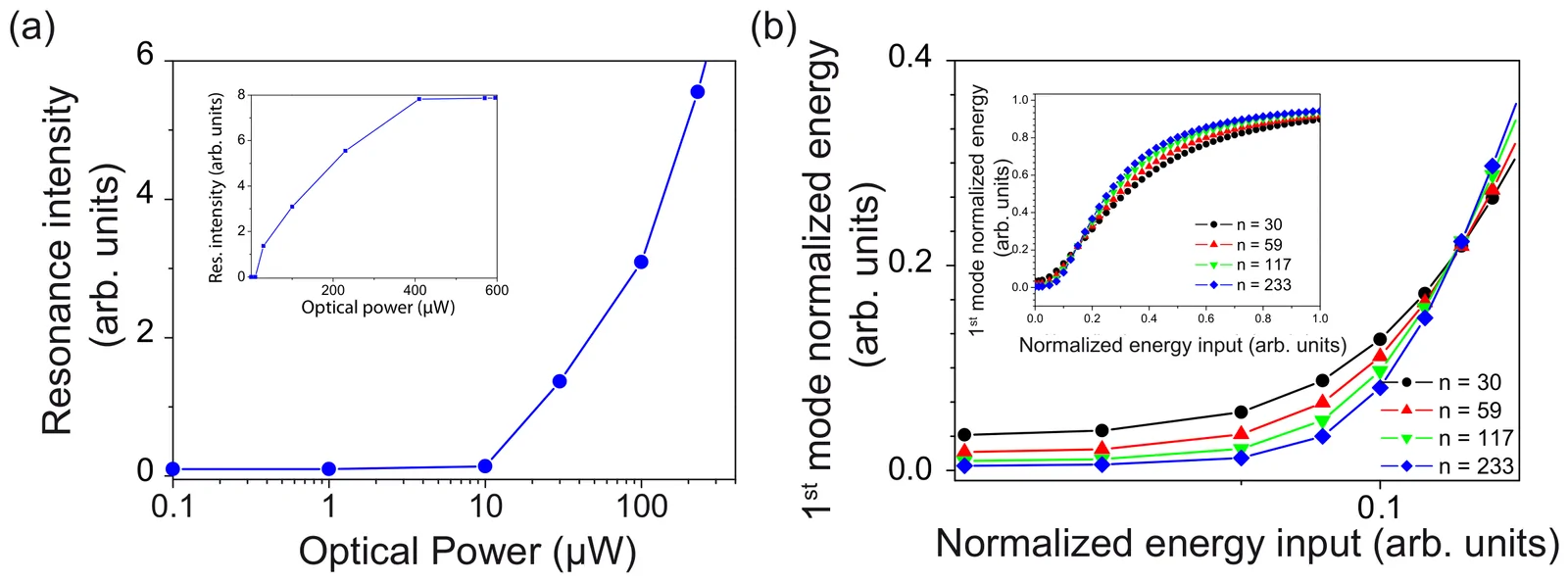
Threshold-like behaviour of giant dipolar oscillations. (**a**) Intensity of the resonant peak measured at 0.314 THz as a function of the optical laser power. (**b**) Normalized energy of the fundamental mode calculated as a function of the normalized source power. The different curves correspond to the reported numbers of normal modes of the BSA protein. Theory and experiment are in *qualitative* agreement. From Ref. [10].

The threshold and saturation behaviours of the resonance intensity, as functions of laser power, match the theoretical predictions from the nonlinear rate Equation (15) (see Figure 11).

A consistency estimate of the onset timescale (several minutes of illumination before the resonance reaches its final amplitude) can be obtained from the energy balance

(40) $$\frac{dE}{dt}=-\frac{2}{3}\frac{{\left(Ze\right)}^{2}{\left|\ddot{x}\right|}^{2}}{c^{3}}-\Gamma +W,$$

where *W* is the optical energy input rate and the first term on the right is the bremsstrahlung (radiative) loss. With five to six Alexa 488 molecules per BSA, each receiving roughly 120–300 photons per second at 500 μW of laser power, the upper bound for *W* is 3.8–$9.5\times 10^{-11}$ erg s^−1^, almost coinciding with the estimated radiative loss of $3.25\times 10^{-12}$–$1.7\times 10^{-11}$ erg s^−1^ [10]. Because the two rates are so nearly balanced, energy accumulates slowly in each molecule, and several minutes elapse before the condensation threshold is crossed. Once the condensed phase is established, the collective low-frequency oscillation carries a very large effective dipole moment: requiring the bremsstrahlung losses of the condensed oscillation to be balanced by *W* implies an oscillating dipole in the range 14,500–23,000 Debye, corresponding to an effective *Z* ≈ 290–460 elementary charges [10]. This is fully consistent with the strong THz absorption feature emerging against the water background (≈2000 dB/cm extinction).

Finally, we note the biological relevance of these estimates. In living cells, a protein molecule undergoes roughly $10^{6}$ collisions per second with ATP molecules at a standard intracellular concentration of ∼1 mM. If even 1–2% of those collisions transfer the ≈50 kJ/mol of ATP hydrolysis free energy to the protein, the available power is ∼$8\times 10^{-9}$–$1.6\times 10^{-8}$ erg s^−1^ per molecule, at least two orders of magnitude larger than what was delivered by the laser in the in vitro experiment in [10]. Phonon condensation in vivo could therefore be activated on a far shorter timescale than the minutes required in the laboratory.

### 8.3. Phonon Condensation in R-PE

R-phycoerythrin (R-PE), a hexameric light-harvesting protein of molecular weight 240 kDa derived from red algae, provides a particularly clean experimental system because it absorbs strongly at 488 nm through its 38 endogenous phycoerythrin and phycourobilin fluorochromes, without requiring external labelling [15]. Under laser illumination with a 488 nm source, a collective oscillation mode appears at $\nu_{0}=71$ GHz (2.4 cm^−1^), displaying both a threshold in the energy input rate and a saturation of the oscillation amplitude at high power, in full analogy with the BSA observations [10]. The collective mode frequency is consistent with the lowest extension mode of a torus of major radius R=37.5 Å and minor radius r=30 Å, yielding a Young’s modulus of E≈5.3 GPa by inversion of the Blevins formula [15], a value that lies squarely between those of myoglobin (3.5 GPa) and BSA (6.75 GPa), both predominantly α-helical proteins like R-PE.

**Figure 12:**
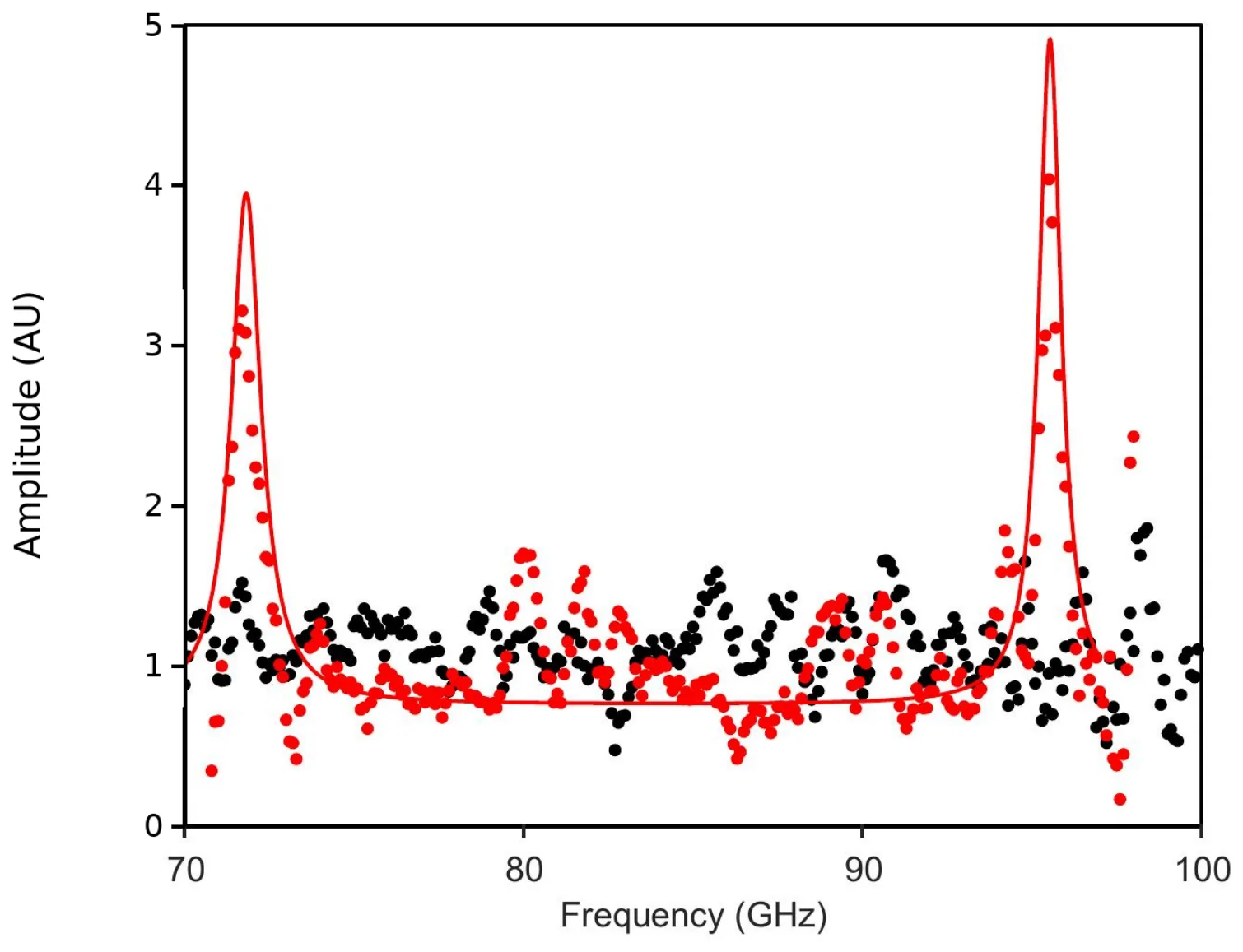
R-PE-coherent vibrational states. Comparison of the R-PE in saline solution (red) and saline solution without R-PE (black). Two collective extension modes of R-PE appear at 71 GHz and 96 GHz. Experimental data (full circles); Lorentz fit (solid line). AU, arbitrary unit. From Ref. [15].

Two collective extension modes appear at 71 GHz and 96 GHz, with the frequency ratio 96/71≈1.35 matching the torus-mode theoretical ratio $\sqrt{2}\approx 1.41$ within 4%. (see Figure 12).

The two-mode structure carries an important mechanistic signature of phonon condensation. The second mode at 96 GHz is observable only after the first mode at 71 GHz has reached saturation [15]. This is precisely what the nonlinear rate equations predict: the condensation mechanism channels all injected energy into the fundamental mode first; only once that mode is saturated does energy become available to excite the next collective mode. In the in vitro experiment, the onset time for the 71 GHz peak is about 1 min (at 50 mW), while approximately 15 min of pumping is required to excite the 96 GHz peak. This hierarchy of timescales is fully consistent with the condensation picture and constitutes an additional internal consistency check on the interpretation. The R-PE experiments do use a substantially higher laser power than the BSA ones, but not because R-PE is a less efficient absorber; if anything, its 38 endogenous chromophores make it considerably more efficient than the 5–6 exogenous Alexa 488 labels attached to BSA. The higher power instead reflects a difference in experimental requirements: the FCS clustering measurement (Section 8) requires the collective oscillation to be established and the resulting electrodynamic force to reach a magnitude sufficient to produce a measurable *D*/*D*_0_ drop within the time a molecule spends transiting the confocal volume, whereas the BSA THz measurement uses a static drop under long, low-intensity illumination with no such time constraint (onset over several minutes, Section 8). The 50 mW drive is thus better read as the power needed to reach condensation and activate a strong enough $1/r^{3}$ interaction on a suitably short timescale, not as compensation for inefficient absorption; we agree this power regime is at the high end of what metabolic sources alone would supply in vivo and should be read as a controlled, artificially strong drive used to bring the clustering signature within reach of the FCS protocol, rather than as a claim about physiological power densities. On possible photothermal artefacts: a uniform temperature rise would shift the Brownian diffusion coefficient *D*_0_ smoothly and monotonically with laser power and concentration, whereas the observed effect is a sharp, threshold-like drop in *D*/*D*_0_ at a specific intermolecular distance, together with full reversibility on a timescale of seconds after the laser is switched off (Section 8); neither feature is expected of bulk heating, local convection, or micro-boiling, which would not be expected to reverse on this timescale or to depend on intermolecular distance in this way. A direct, independent measurement of the local temperature rise under 50 mW illumination (e.g., via a temperature-sensitive fluorescent reporter) was not performed in Ref. [15] and is noted here as a useful control for future work.

### 8.4. Clustering Phase Transition

**Figure 13:**
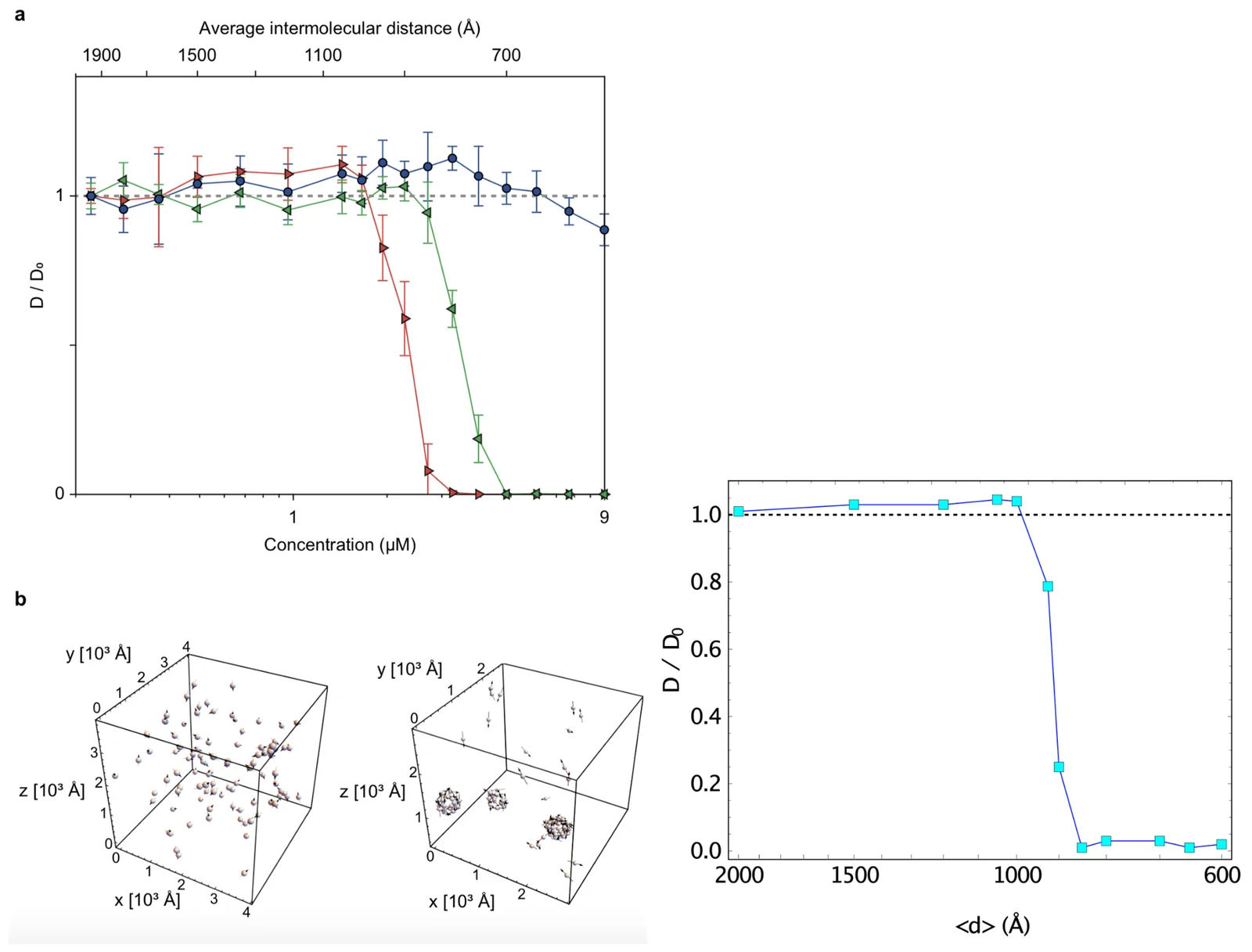
Effect of protein concentration and laser power illumination on R-PE diffusion: Clustering phase transition. (**a**) Diffusion coefficients normalized to the Brownian $D_{0}$ values measured for each data series at 0.223 μM (〈r〉≃1950 Å) and recorded at 50 μW (blue circles), 100 μW (green triangles), and 150 μW (red triangles). Each point corresponds to the average of 5 independent experiments. (**b**) Clustering transition driven by long-range electrodynamic interparticle forces, obtained from Monte Carlo simulations by varying the initial mean separation 〈r〉. For ${\langle r\rangle }_{\mathrm{init}}≃1000$ Å the system remains in the dispersed phase ($D/D_{0}=1$, Brownian diffusion). For ${\langle r\rangle }_{\mathrm{init}}≃950$ Å it switches to the clustered phase ($D/D_{0}\ll 1$). (**Lower right**) panel: Molecular Dynamics computation of self-diffusion coefficient $D/D_{0}$ (normalized to the Brownian value $D_{0}$) versus intermolecular average distance 〈d〉 for a system of particles in a cubic box interacting through long-range electrodynamic forces. From Ref. [15].

The most striking macroscopic consequence of the activated electrodynamic forces is a sharp, discontinuous-looking clustering transition in R-PE solutions, detected by fluorescence correlation spectroscopy (FCS) and described in Ref. [15] as first-order-like in character, on the basis of the abruptness of the *D*/*D*_0_ drop and the existence of a well-defined critical intermolecular distance, rather than from a measured thermodynamic hysteresis loop, which was not part of the original protocol. The normalised diffusion coefficient *D*/*D*_0_ measured by FCS is constant across all concentrations at 50 μW (below threshold): a purely Brownian diffusion. At 100 μW and 150 μW, *D*/*D*_0_ drops by several orders of magnitude at average intermolecular distances of ≈725 Å and ≈925 Å respectively. The transition point shifts to larger distances with increasing power (stronger oscillations, stronger forces), and is fully reversible upon switching off the laser. (see Figure 13).

The quantitative extent of the transition is remarkable: at an average intermolecular distance of 600 Å, the variance in fluorescence fluctuations increases by more than five orders of magnitude compared with the same quantity at 1950 Å, and the measured diffusion time $\tau_{D}$ jumps from $4.8\times 10^{-5}$ s to 4.9 s, a factor of ∼$10^{5}$ [15]. These values cannot be explained by any trivial heating effect: a thermal increase in diffusivity would shift $D_{0}$ uniformly and monotonically with concentration, producing no step-like feature. The molecular dynamics simulations with a $1/r^{3}$ interaction potential reproduce the measured critical distance at 150 μW quantitatively, providing independent theoretical support for the experimental interpretation. Monte Carlo computations confirm the formation of dense clusters below the critical intermolecular distance [15]. These are standard Metropolis Monte Carlo simulations in the canonical (NVT) ensemble: point-like particles interacting through the resonant $1/r^{3}$ electrodynamic potential of Equation (22) (sign and magnitude set by the same action difference, $J_{i,+}-J_{i,-}$, introduced in Section 5) are placed in a cubic box with periodic boundary conditions, at particle numbers and box sizes chosen to reproduce the experimental protein concentrations, and equilibrated over a number of Monte Carlo sweeps sufficient for the pair correlation function to stabilise. The full parameter set (particle number, box size, interaction cut-off, and number of equilibration/sampling sweeps) is reported in the Supplementary Information of Ref. [15]; we have added an explicit link to it here. Direct visual evidence was obtained by confocal fluorescence microscopy: clusters of R-PE molecules appear and grow when the laser power exceeds the threshold, then rapidly dissolve when the laser is switched back to a power below the threshold for activating collective oscillations [15]. This reversibility on a timescale of seconds rules out irreversible photochemical aggregation and is fully consistent with the dynamical, energy-dependent nature of the underlying ED forces.

The fact that BSA does not show the diffusion-based clustering signal is itself informative and consistent with the theory. As discussed above, BSA requires approximately 10 min of optical pumping to activate a sharp collective oscillation. In FCS geometry, the transit time of each protein through the confocal volume is far shorter than this activation time, so the collective oscillations cannot be established during the measurement. By contrast, R-PE, with its 38 endogenous fluorochromes and natural light-harvesting efficiency, reaches a condensed state in less than 3 s under comparable power density, making FCS measurement of its clustering feasible [15]. BSA is instead uniquely suited to the THz frequency-shift measurement, which uses long-exposure illumination of a static drop of solution rather than molecules in transit. Thus, the two proteins play complementary roles and together constitute a self-consistent body of evidence.

These experimental outcomes are in quantitative agreement with the theoretical prediction of a clustering transition driven by the resonant $-1/r^{3}$ electrodynamic interaction potential, activated out of thermal equilibrium [15].

### 8.5. Frequency Shifts: Further Evidence for Long-Range Electrodynamic Forces

If resonant electrodynamic forces are active between collective dipoles oscillating at a frequency of $\omega_{0}$, the vibration frequency of each molecule shifts by $\Delta \nu \propto 1/{\langle r\rangle }^{3}\propto C$ (concentration), where $\langle r\rangle =C^{-1/3}$ is the average intermolecular distance [11,15]. This prediction is confirmed for both R-PE (at 71 GHz) and BSA (at 0.314 THz): the measured frequency shift varies linearly with concentration, with a slope that increases with laser power (stronger oscillation amplitude implies larger dipole moment and hence stronger interaction). Experiments were performed in 200 mM NaCl solution, ensuring Debye screening of all electrostatic interactions.

**Figure 14:**
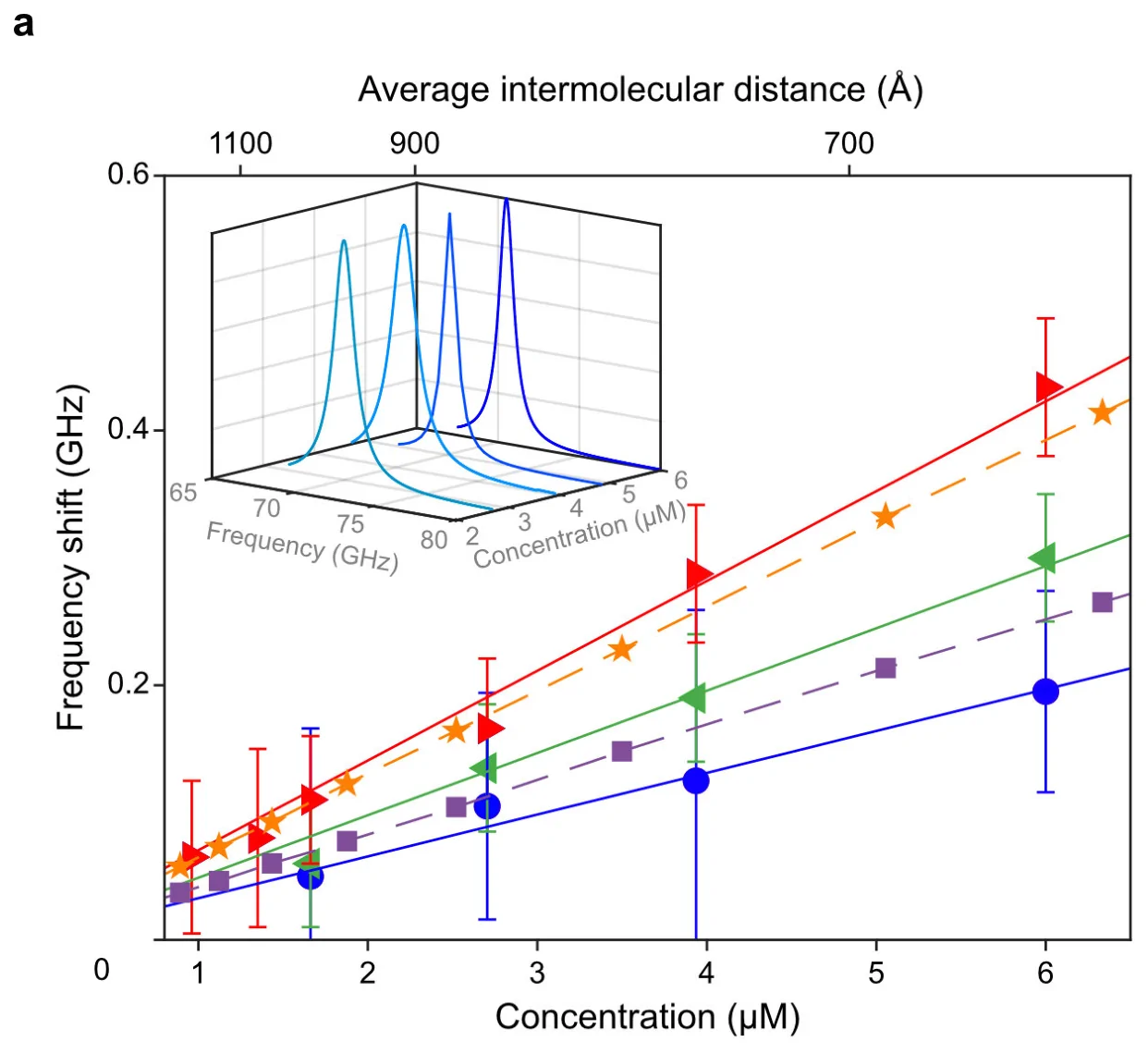

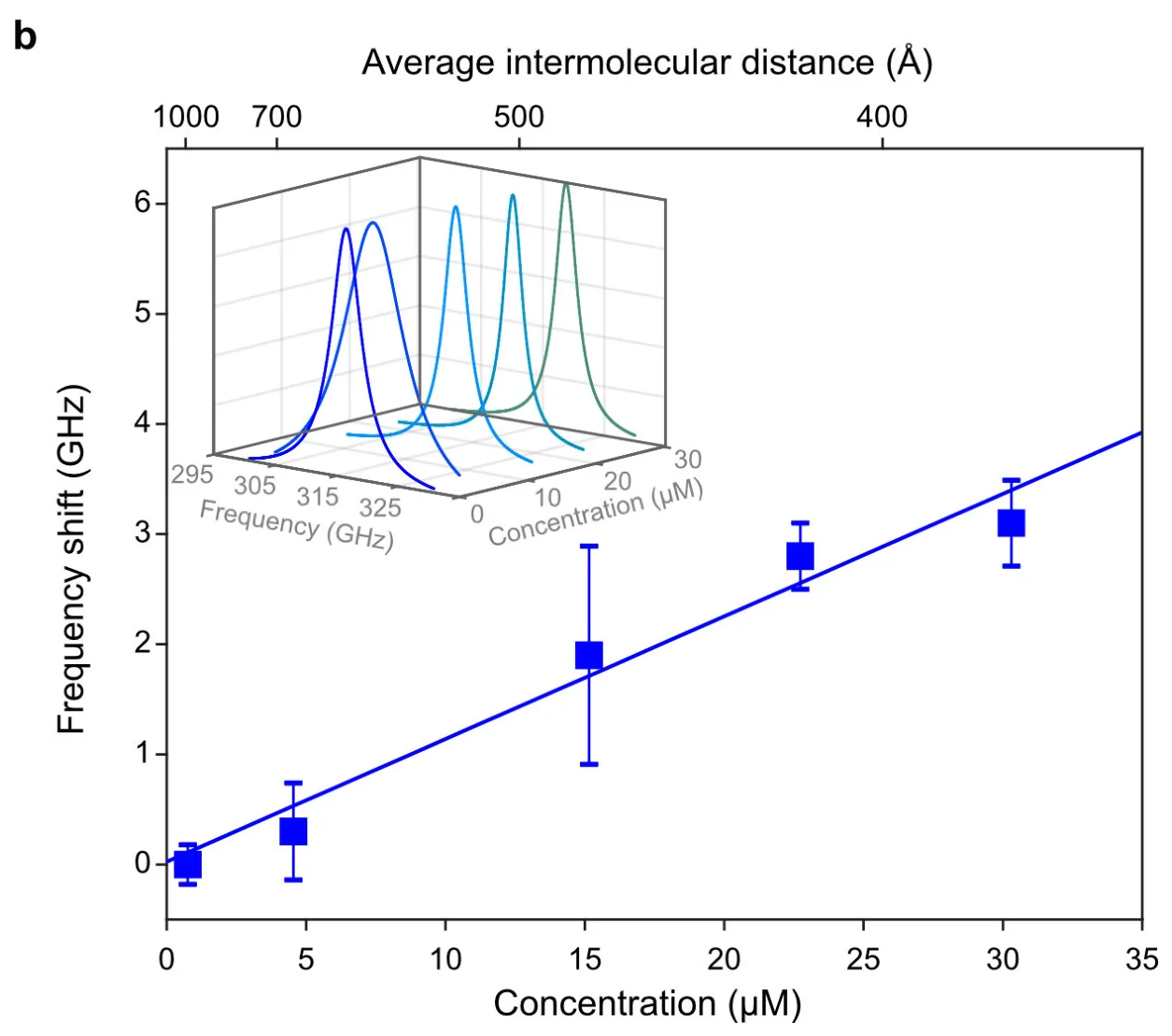
Frequency shifts of the intramolecular collective vibrations of R-PE and BSA at different concentrations. Measurements were performed at room temperature in aqueous solution with 200 mM of NaCl. Panel (**a**) refers to R-PE. The shift is relative to the reference frequency measured at the lowest protein concentration. Measurements have been performed at the different powers of the laser: 31.5 mW (blue circles), 39.5 mW (green triangles), and 50 mW (red triangles). Purple squares and orange stars refer to theoretical outcomes worked out with different values of molecular dipole moments (see Supplementary Materials of Ref. [15]). Panel (**b**) refers to BSA at a laser power of 40 mW. Insets: The frequency shifts are measured through a Lorentz fitting of the experimental resonances. The different colours in the insets are just a visual aid. From Ref. [15].

For R-PE, frequency-shift measurements were performed at three laser powers: 31.5, 39.5, and 50 mW. In all cases, the shift is linear in concentration, confirming the $1/{\langle r\rangle }^{3}$ dependence over the range of average intermolecular distances from roughly 700 to 1100 Å explored in the THz experiments [15]. The steepening of the slope with laser power is also quantitatively consistent with theory: a larger oscillation amplitude generates a larger oscillating dipole moment and hence a stronger electrodynamic coupling. The BSA frequency-shift measurement (at 40 mW) confirms the same linear law at 0.314 THz, extending the evidence for long-range electrodynamic forces to a structurally unrelated protein. (see Figure 14). Crucially, the THz spectroscopy setup for these frequency-shift measurements used a lower spatial density of laser light than the FCS diffusion experiments, specifically to prevent a clustering transition during the measurement: the ED forces were deliberately weakened to remain below the threshold for cluster formation, so that the proteins kept diffusing freely while still experiencing the frequency shift [15]. This experimental design decouples the two observable signatures of ED forces and rules out the cross-contamination of the two measurements.

## 9. Discussion and Outlook

A distinctive feature of the theoretical framework reviewed here is that the TDVP serves as a single unifying instrument across different physical scenarios. In Section 3, applied to product-coherent states of bosonic operators, it produces a classical Hamiltonian in action-angle variables directly suitable for statistical (Liouville/KvN) analysis. In Section 7, applied to the Davydov factorised ansatz of electronic and phononic coherent states, it produces classical equations of motion for electron probability amplitudes coupled to classical phonon degrees of freedom, encoding the specific biomolecular sequence at the level of site-dependent coupling constants. In both cases, the resulting dynamics are formally classical but directly inherit the quantum structure of the original Hamiltonian, without invoking any semi-classical approximation beyond the mean-field restriction to the chosen ansatz manifold.

### 9.1. Two Complementary Channels of Selective Long-Range Forces

The review presents two physically distinct but mathematically parallel channels through which selective long-range electrodynamic interactions can be activated between biomolecules:

*The vibrational (phonon) channel*: Driven by metabolic or photonic energy input, a macromolecule undergoes classical Fröhlich condensation, concentrating energy into a coherent collective oscillation. The resulting giant oscillating dipole moment generates $1/r^{3}$ resonant forces, confirmed experimentally by THz spectroscopy and FCS.

*The electronic channel*: Electron–phonon excitation along a specific DNA sequence or protein produces oscillating electron currents whose Fourier spectrum carries the “fingerprint” of the biomolecular sequence. The cross-spectrum of two interacting molecules displays a sharp co-resonance peak when and only when their sequences are cognate, providing a sequence-specific electrodynamic interaction mechanism.

The two channels operate at different energy and frequency scales (sub-THz vs. tens of THz) and are not mutually exclusive; in a living cell, they may well operate simultaneously and synergistically.

### 9.2. Open Questions

#### 9.2.1. From Light Pumping to ATP

The experiments use laser excitation, which is a controlled but artificial energy supply. ATP hydrolysis provides an estimated power of ∼$10^{-8}$–$10^{-7}$ erg s^−1^ per protein [10], orders of magnitude larger than the laser power available per protein in the experiments. The position-space Hamiltonian framework [12], via normal-mode analysis of force fields, offers a path toward modelling ATP-coupled condensation in atomistic detail. The comparison in Section 8 is intended as an order-of-magnitude plausibility argument, not a claim that a non-enzymatic protein such as BSA captures ATP hydrolysis energy the way the Alexa 488 fluorochrome captures photons. In the laser experiment, the transducer is explicit and controlled: the dye’s electronic excitation decays non-radiatively into the protein’s vibrational modes. In vivo, BSA itself has no ATPase domain and is not proposed as a site of direct ATP capture; the relevant biological transducers would instead be the proteins that *do* couple mechanically to ATP hydrolysis, molecular motors, ATP-driven ion pumps, and enzymes undergoing hydrolysis-linked conformational cycling (the repeated cycle of structural changes many ATP-driven enzymes undergo per catalytic turnover), whose conformational energy is already known to excite low-frequency collective modes of the protein itself and, potentially, of non-catalytic neighbours coupled to it elastically or electrodynamically. Establishing a concrete, ATPase-mediated microscopic pathway that funnels hydrolysis energy into a Fröhlich-like collective mode, as opposed to the photonic pathway reported here, remains an explicit target for future work rather than a mechanism demonstrated in the hitherto available experiments.

#### 9.2.2. Role of Water

The hydration shell plays an active, and mechanistically distinct, role in each channel. In the vibrational (phonon) channel (Section 5), the surrounding ionic solution first removes the static Debye screening above ∼250 MHz that would otherwise mask any dipole–dipole interactions, and then, through the frequency-dependent permittivity ε(ω), sets the effective range and damping of the resonant $1/r^{3}$ term itself; water is thus a necessary enabling medium for this channel rather than a passive background medium. In the electronic (DNA–protein) channel (Section 7), by contrast, the relevant role of water is protective rather than propagating: the collective, coherently reorientable dipoles of the surrounding water molecules couple to the electromagnetic field radiated by the DNA’s own oscillating electron current, quantized as a single bosonic mode in the Jaynes–Cummings-like Hamiltonian of Section 7, through a coupling whose strength scales as $\sqrt{N}$ in the number of participating water dipoles [13,31], providing a cooperative Tavis–Cummings-type shield against thermal decoherence for the electronic correlations described in Section 7. A quantitative theory connecting these two distinct roles of the aqueous environment within a single, unified treatment of the water degrees of freedom remains to be developed.

#### 9.2.3. Predictive Power for Mutations and Conservation

In its present form, the model is diagnostic rather than predictive: given a candidate sequence and its recognition partner, it computes whether the electron–phonon co-resonance is present or degraded, as illustrated here for a handful of point mutants with known biochemical phenotype (Section 7). It does not yet output an a priori probability that a given mutation will be deleterious, nor has it been tested against the statistics of naturally conserved versus variable sites in curated genomic or mitochondrial variant databases. Doing so would require applying the same electron–phonon Hamiltonian systematically across many sequence contexts and comparing the resulting co-resonance strength to independent measures of conservation or pathogenicity, a large-scale computational undertaking that the present review flags as an open direction rather than reporting it as completed work.

#### 9.2.4. In Vivo Evidence

Whether these phenomena operate in the complex, crowded, and dynamically heterogeneous cellular environment is the central open question. Selectivity of electrodynamic forces in a mixture of many molecular species, the role of molecular crowding, and the competition between ED forces and Brownian diffusion all require further investigation. Crowding is expected to affect the in vivo signal in two distinct ways that the present in vitro measurements cannot separate. First, the frequency selectivity of the resonant interaction (Section 5) is itself a form of protection against non-resonant neighbours: because the near-resonance $1/r^{3}$ term collapses to a short-range $1/r^{6}$ potential away from resonance, non-cognate proteins vibrating at other frequencies are not expected to couple strongly or to absorb at the collective mode’s own resonance frequency (e.g., 0.314 THz for BSA, via Equation (39), or the 71/96 GHz doublet of R-PE, Section 8), a frequency set by each macromolecule’s own size and elastic modulus, so that non-cognate neighbours should contribute background dielectric loss rather than resonant quenching. Second, and independently of resonance, the sheer optical density of a crowded cytoplasm at sub-THz frequencies could still damp or scatter the field non-resonantly, reducing the effective interaction range compared with the dilute in vitro solutions studied here; the magnitude of this non-resonant background loss in a physiologically crowded medium has not yet been measured and is an explicit target for future in-cell THz spectroscopy.

#### 9.2.5. Quantum Corrections

The classical framework is quantitatively justified at sub-THz frequencies and room temperature, but quantum fluctuations in the condensed state [33] and quantum coherence in the electronic channel may be non-negligible and merit further study.

#### 9.2.6. Connection to Biomolecular Condensates

The first-order clustering transition driven by electrodynamic forces provides a physically distinct mechanism for the formation of reversible biomolecular assemblies [1], complementing models based on intrinsically disordered regions and multivalency. Whether this mechanism is relevant to phase-separated cellular compartments is an open and intriguing question. A direct test of the first-order character invoked above would be to ramp the laser power up and down slowly across the transition and look for a hysteresis loop in $D/D_{0}$ (or in the clustered-fraction order parameter) as a function of power; such a protocol was not part of the original measurements and is proposed here as a concrete next experiment.

### 9.3. Concluding Remarks

The programme reviewed here demonstrates that classical Hamiltonian mechanics, derived systematically from quantum models via the TDVP and implemented at the level of both rate equations and direct Hamiltonian simulation, produces rich and experimentally verifiable physics relevant to the organisation of biochemical reactions in living matter. Fröhlich’s half-century-old intuition has acquired rigorous Hamiltonian underpinning, direct experimental support, and a new electronic dimension through the DNA–protein co-resonance phenomenology. The next challenge is to establish whether and how these phenomena operate in the cellular environment, and what role they play in the extraordinary efficiency of biological molecular recognition. More broadly, the results reviewed here suggest that the nonequilibrium collective states of biological macromolecules are not merely a theoretical curiosity but a measurable and controllable physical phenomenon, with a growing toolkit, including THz near-field spectroscopy, fluorescence correlation spectroscopy, and confocal imaging of clustering, for probing it directly. If the vibrational and electronic channels described here indeed operate together in living cells, they would offer a physically grounded, sequence- and state-dependent selectivity mechanism that complements, rather than replaces, standard biochemical specificity, with potential relevance to how metabolic state modulates molecular recognition, and to the biophysical underpinnings of biomolecular condensates (liquid–liquid phase separation) more generally. Turning this possibility into a quantitative, in-cell research programme, extending the position-space Hamiltonian framework to realistic force fields, quantifying the water contribution, and testing the electronic co-resonance mechanism against genomic-scale sequence data, is, in our view, the most promising direction for the field in the near term.

## Data Availability

No new data were created or analyzed in this study. Data sharing is not applicable to this article.
